# Dual and opposing roles of the EXD2 exonuclease in the resolution of RNA–DNA hybrids

**DOI:** 10.1093/nar/gkag762

**Published:** 2026-07-31

**Authors:** Meng Hu, Yinghong Chen, Yanan Li, Xia Zhang, Yi Zhao, Xiaoxuan Song, Lishuang Chen, Zhiyun Ren, Yuting Zhang, Yali Mi, Cong Liu, Wei Li, Chao Liu, Bo Sun

**Affiliations:** School of Life Science and Technology, ShanghaiTech University, Shanghai 201210, China; Guangzhou Women and Children’s Medical Center, Guangzhou Medical University, Guangzhou, Guangzhou 510623, China; Department of Basic Medical Sciences, Shantou University Medical College, Shantou, Guangdong 515041, China; School of Life Science and Technology, ShanghaiTech University, Shanghai 201210, China; College of Life Sciences, Xinyang Normal University, Xinyang, Jinan 464000, China; School of Life Science and Technology, ShanghaiTech University, Shanghai 201210, China; School of Life Science and Technology, ShanghaiTech University, Shanghai 201210, China; School of Life Science and Technology, ShanghaiTech University, Shanghai 201210, China; School of Life Science and Technology, ShanghaiTech University, Shanghai 201210, China; School of Life Science and Technology, ShanghaiTech University, Shanghai 201210, China; CAS Center for Excellence in Molecular Cell Science, Shanghai Institute of Biochemistry and Cell Biology, Chinese Academy of Sciences, Shanghai 200031, China; College of Life Sciences, University of Chinese Academy of Sciences, Beijing 100049, China; School of Life Science and Technology, ShanghaiTech University, Shanghai 201210, China; Guangzhou Women and Children’s Medical Center, Guangzhou Medical University, Guangzhou, Guangzhou 510623, China; Interdisciplinary Research Center on Biology and Chemistry, State Key Laboratory of Chemical Biology, Shanghai Academy of Natural Sciences (SANS), Shanghai Institute of Organic Chemistry, Chinese Academy of Sciences, Shanghai 201210, China; Guangzhou Women and Children’s Medical Center, Guangzhou Medical University, Guangzhou, Guangzhou 510623, China; Guangzhou Women and Children’s Medical Center, Guangzhou Medical University, Guangzhou, Guangzhou 510623, China; School of Life Science and Technology, ShanghaiTech University, Shanghai 201210, China

## Abstract

Nucleases are specialized enzymes known for degrading nucleic acids in diverse cellular processes. Among them, EXD2 contributes to genome maintenance by digesting a broad range of nucleic acid substrates, including non-canonical RNA–DNA hybrids (RDHs). However, the molecular mechanism underlying the interplay of EXD2 with these hybrid structures has remained elusive. Here, using an optical tweezers-based single-molecule approach, we unveil that EXD2 cooperatively binds to and slowly digests mechanically tensioned RDHs. On the other hand, the cooperative binding of a high amount of EXD2 onto a relaxed RDH drives their co-condensation. Moreover, EXD2 is capable of recognizing damage sites along RDHs and inducing damaged RDH condensation even at low protein concentrations. Surprisingly, this co-condensation, in contrast, protects RDHs from timely degradation by other nucleases. Consistently, we unveil that overexpressed EXD2 colocalizes with mitochondrial RDHs and, rather than cleavage, prevents them from fast degradation. Therefore, EXD2 is a promiscuous moonlighting enzyme that can exert opposing activities toward RDHs. Our findings provide a mechanistic understanding of the functional roles of EXD2 in cells.

## Graphical Abstract

**Graphical Abstract:**
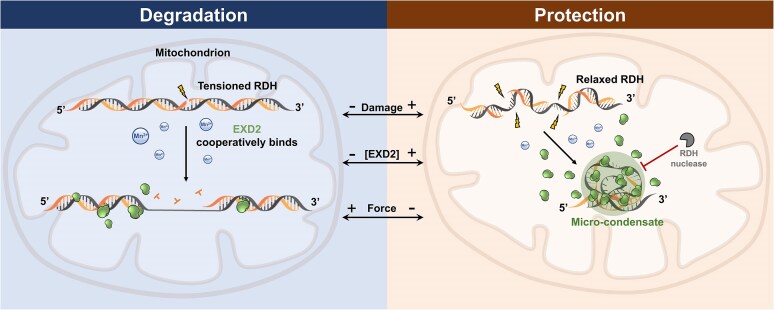

## Introduction

Enzymes are traditionally regarded as highly specific catalysts, each evolved to perform a defined biochemical function with high fidelity under physiological conditions [1–3]. Nevertheless, this classical paradigm is increasingly challenged by the phenomenon of enzyme promiscuity, wherein a specialized protein possesses the latent capacity to catalyze “side” reactions in addition to its native function [4–7]. Such promiscuous activities represent a “plastic” functional repertoire that enables organisms to adapt to varying environmental conditions and molecular contexts [5, 8, 9]. For instance, conditional promiscuity allows an enzyme to toggle its catalytic behavior or substrate processing mode in response to cellular stressors or physical cues [9]. Notably, some enzymes have even evolved to possess non-canonical functions and are referred to as moonlighting enzymes [10, 11]. Importantly, this inherent promiscuity provides a molecular starting point for functional diversification, driving the evolutionary adaptability required to acquire novel biological roles while maintaining native protein stability [6, 7]. In the context of nucleic acid metabolism, such promiscuous enzymes frequently operate at the interface of genome maintenance and regulatory control, enabling dynamic, context-dependent responses to changing cellular states [5, 7, 12].

Nucleases are a group of specialized enzymes that catalyze the cleavage of phosphodiester bonds to process, remodel, or remove nucleic acids [13–15]. These enzymes are indispensable in nearly all aspects of nucleic acid metabolism, such as DNA recombination and RNA translation [16–18]. EXD2, a recently identified nuclease, was initially characterized as a mitochondria-associated enzyme required for mitochondrial DNA (mtDNA) repair and RNA translation [19, 20]. However, emerging evidence indicates that EXD2 is a highly mobile enzyme that redistributes between the mitochondrial outer membrane, the mitochondrial interior, and the nucleus [19–24]. Besides the functional roles in mitochondria, EXD2 also contributes to the maintenance of genome stability in the nucleus through DNA resection in double-strand break (DSB) repair and protection of stressed replication forks [20, 25–27]. Remarkably, tasked by these multiple cellular functionalities, EXD2 is capable of resolving not only traditional DNA and RNA structures but also hybrid nucleic acid motifs, such as RNA–DNA hybrid (RDH) and R-loop [19, 20, 28, 29]. From the structural perspective, EXD2 belongs to the DnaQ-like 3′–5′ exonuclease family and harbors a conserved catalytic domain with the hallmark acidic residues for metal ion coordination and exonucleolytic cleavage [21, 30, 31]. This conserved active site serves as a robust engine for phosphodiester bond hydrolysis while its modular architecture allows for versatile substrate accommodation [31]. Consequently, this architecture provides EXD2 with the flexibility to process a diverse range of substrates [32]. At the molecular level, we have revealed that EXD2 resolves RDHs with a preference for degrading RNA strands, and this resolution proceeds through a discrete nucleotide excision mechanism that is tightly coupled to substrate unwinding [33]. Despite these studies, how EXD2 specifically targets and processes hybrid structures, particularly long substrates, remains unclear.

In this study, we investigated the molecular mechanism underlying the interplay between EXD2 and RDHs at the single-molecule and cellular levels. Using single-molecule analysis, we revealed that EXD2 binds RDHs in a positively cooperative manner, and it exerts an extremely slow and nonprocessive exonucleolytic degradation toward tensioned RDHs. However, a sufficient amount of EXD2 proteins aggregating along relaxed hybrids causes their co-condensation; strikingly, in contrast to cleavage, EXD2-induced RDH condensation protects them from fast degradation by other nucleases. Moreover, the presence of damage sites within RDHs further promotes EXD2 binding and thus RDH condensation and protection. *In vivo* validated the colocalization of EXD2’s mitochondrial RDHs, and upon overexpression, it consistently retarded RDH resolution. Therefore, EXD2 is identified as a promiscuous enzyme, capable of switching between opposing degradative and protective modes on a long stretch of RDH substrate in a condition-dependent manner. These findings add a new dimension to enzymatic multi-functionality in genome maintenance.

## Materials and methods

### Protein expression and purification

**EXD2**. Recombinant EXD2 (61–621) was used for protein expression because of its improved solubility and yield [21, 33]. The EXD2 gene was amplified by PCR using *Homo sapiens* complementary DNA as a template and inserted between the SacI and XhoI sites of the plasmid pET28a. In the resulting plasmid, pET28a-EXD2, the N-terminus of EXD2 was fused to a His_6_-tag, which has proved not to interfere with the exonuclease activity of EXD2. The gene encoding EXD2-eGFP, with eGFP fused to the C-terminus of EXD2, was cloned into the same vector. The expression plasmid was transformed into BL21 (DE3) (TransGen). This strain was then cultured and induced by 1 mM isopropyl-1-thio-d-galactopyranoside (IPTG) at 18°C for 16 h. Cells were collected by centrifugation and resuspended in the lysis buffer containing 50 mM HEPES (pH 7.5), 300 mM NaCl, 10 mM imidazole, 5% glycerol, and 1% protease inhibitor cocktail, and passed through a homogenizer three times at ~800 bar. The lysed dilution was then ultracentrifuged at 11 000 × rpm for 30 min. The supernatant was applied to an Ni-NTA resin (TransGen) and was washed extensively with 50 mM HEPES (pH 7.5), 300 mM NaCl, 20 mM imidazole, and 5% glycerol. Then, the bound protein was eluted in a single step with the elution buffer containing 50 mM HEPES (pH 7.5), 300 mM NaCl, 250 mM imidazole, and 5% glycerol. Further purification was performed by a 5 ml Q Sepharose strong anion exchange column (GE Healthcare). Finally, the protein sample was buffer exchanged into the storage buffer containing 50 mM HEPES (pH 7.5), 500 mM NaCl, and 10% glycerol and stored at −80°C before use.

**RNase H1**. The plasmid designed for the expression of Yeast RNase H1 was constructed by synthesizing open reading frames and integrating them into pET28a [34, 35]. The N-terminus of the resulting pET28a-RNase H1 plasmid was fused to a His_6_-tag for purification. *Staphylococcus aureus* sortase A recognition hexa-amino acid sequence (LPETG) was introduced onto the N-terminus of RNase H1 behind the His_6_-tag for protein labeling. The plasmid was transformed into BL21 (DE3) cells (TransGen). This strain was cultured and induced by 1 mM IPTG at 18°C for 16 h. Following that, cells were collected by centrifugation and resuspended in the lysis buffer containing 50 mM Tris–HCl (pH 7.5), 300 mM NaCl, 10 mM imidazole, 5% glycerol, and 1% Protease Inhibitor Cocktail, and passed through a homogenizer three times at ~800 bar. The resulting lysate was ultra-centrifuged at 11 000 × rpm for 30 min, and the supernatant was subjected to Ni-NTA resin (TransGen) affinity purification. This bound protein was extensively washed with a solution containing 50 mM Tris–HCl (pH 7.5), 300 mM NaCl, 30 mM imidazole, and 5% glycerol. The protein was eluted using elution buffer containing 50 mM Tris–HCl (pH 7.5), 300 mM NaCl, 300 mM imidazole, and 5% glycerol. The protein sample underwent chromatography for further purification using a 5-ml HiTrap Heparin HP column. Ultimately, the proteins were buffer-exchanged into a storage buffer containing 50 mM Tris–HCl (pH 7.5), 500 mM NaCl, and 10% glycerol and stored at −80°C. The protein labeling reaction was conducted in 1× sortase buffer containing 50 mM Tris–HCl (pH 7.5), 250 mM NaCl, and 10 mM CaCl_2_ at 4°C overnight with LPETG-tag RNase H1, *S. aureus* sortase A, and the Cy3-CLPETGG (QYAOBIO (ChinaPeptides) at a ratio of 1:2:5. Unreacted-free Cy3-LPETGG was removed using a Centrifugal Filters 30 K (Millipore) with a solution containing 50 mM Tris–HCl (pH 7.5), 500 mM NaCl and 5% glycerol. *Staphylococcus aureus* sortase A was removed through chromatography using a 5-ml HiTrap Heparin HP column. Elution of Cy3-labeled RNase H1 occurred around 400~500 mM NaCl, yet the sortase A did not bind to heparin. The labeling efficiency of RNase H1 was typically around 70%. Purified Cy3-labeled RNase H1 was then stored at −80°C.

### Preparation of RNA–DNA hybrid templates

#### Biotinylated 12.3-kbp RDH

The RDH templates used in the optical tweezer assays were prepared through the following steps [35, 36]. The template typically consists of a two-sided biotinylated single-stranded (ss)DNA and a paired ssRNA. A dsDNA segment was PCR-amplified from the lambda DNA by FastPfu Fly DNA Polymerase (TransGen Biotech). Using this dsDNA segment and a biotin-labeled primer, a second asymmetric PCR produced the ssDNA (Supplementary Table S1). The resulting dsDNA and ssDNA were separated by agarose gel electrophoresis and purified using GeneJET Gel Extraction Kit (Thermo). The 3′ end biotin labeling of the ssDNA was performed using biotin-dATP (Invitrogen) and terminal transferase (NEB) (Supplementary Table S1). To construct the ssRNA, a 12.3-kbp dsDNA including a T7 promoter (T7 DNA) at the 5′ end was PCR-amplified from λ phage DNA (Supplementary Table S1). The 12.3-knt ssRNA was generated by transcribing from the T7 DNA. The final RDH templates were produced by annealing the two single-stranded nucleic acids in the TE buffer.

#### Biotinylated nicked/gapped 12.3-kbp RDH

The procedures are similar to those of the RDHs. ssRNA was transcribed from two pieces of dsDNA (8 kbp and 4.3 kbp), and they are paired with two regions of the 12.3-knt ssDNA, respectively. The nicked hybrid templates were generated by annealing products of the ssDNA and its paired two pieces of ssRNA.

### Fluorescence-combined optical tweezers assays

#### EXD2 binding RDH assays

Single-molecule optical-tweezer assays were performed at 25°C on an instrument combining two-colour confocal fluorescence microscopy with dual optical traps (LUMICKS C-trap, Netherlands). The separation between the traps was controlled using a piezo mirror to steer one of the traps. Force measurements were conducted by back-focal plane interferometry of the condenser top lens. The trap stiffness was typically set around 0.6 pN/nm and calibrated by measuring the effect of the optical trap on the Brownian motion of the trapped microsphere [37]. A computer-controlled stage facilitated the rapid movement of the optical traps within a multiple-channel flow cell. This microfluidic system enabled the swift *in situ* construction and characterization of RDH dumbbell constructs and allowed for the efficient transfer of tethered RDH between different flow channels. In the assay, an RDH molecule was initially captured between two streptavidin-coated polystyrene beads (1.76 μm in diameter, Spherotech). This hybrid tether was then transferred to the EXD2 channel. A single RDH molecule was confirmed by its inherent mechanical force–extension relationship. After the confirmation, the RDH tether was transported to the protein or buffer channel, as described for each assay. During data acquisition, a controlled optical trap high-frequency feedback system was used to apply a force (~10 pN) to the RDH tether structure, fixing its distance at 3.6 μm. Unless otherwise specified, all experiments were performed in a reaction buffer containing 25 mM Tris–HCl (pH 7.5), 100 mM NaCl, 1 mM MnCl_2_, 0.05 mg/ml bovine serum albumin (BSA), and 1 mM dithiothreitol (DTT). For EXD2-eGFP imaging, a 488 nm excitation laser was employed, the confocal pixel size was set at 75 nm, and the pixel dwell time was 50 ms.

#### EXD2-catalyzed RDH cleavage assays

The 12.3-kbp RDH substrate tethered between two streptavidin-coated microspheres was transferred to the channel containing EXD2 at the indicated concentrations. During data acquisition, different constant forces were applied to the RDH tether structure via the high-frequency feedback system of the controlled optical trap.

#### EXD2-mediated RDH condensation and stretching assays

During data acquisition, unless otherwise specified, a constant force (~0.10 ± 0.08 pN) was applied to the RDH tether structure via the high-frequency feedback system of the controlled optical trap. The RDH was then transferred into channels containing proteins at different concentrations or buffer only, according to experimental requirements. In the RDH relaxation and stretching assays, the RDH was stretched at a constant rate of 0.1 μm/s [37, 38].

#### EXD2 binding and condensing damaged RDH assays

Intact 12.3-kbp RDH templates were captured between two streptavidin-coated polystyrene beads, and their mechanical integrity was verified via force–extension profiling. The captured RDH was then transferred into a reaction channel containing 5 nM RNase H1 for 5 s to introduce internal RNA nicks/gaps while maintaining the overall structural continuity of the hybrid tether. Following this transient incubation, the “nicked” RDH template was immediately moved into the EXD2-eGFP protein channel for real-time fluorescence detection. The protein-binding dynamics were first monitored by holding the trap-to-trap distance constant at 3.6 μm (Fig. 5A). The template was transferred and maintained under a constant tension of 0.1 pN using an automated force-feedback loop (Fig. 5C). These experimental regimes allowed for the systematic characterization of EXD2 recruitment and assembly on RDH templates populated with multiple RNA gaps. The reaction buffer in the RNase H1 channel consisted of 25 mM Tris–HCl (pH 7.5), 50 mM NaCl, 1 mM MgCl_2_, 0.05 mg/ml BSA, and 1 mM DTT. The reaction buffer used in the EXD2 channel was identical to that used in the other EXD2 binding experiments.

### Single-molecule data analysis

#### Fluorescence intensity analysis

The single-molecule data were analyzed using custom software provided by LUMICKS. Fluorescence intensities of EXD2-eGFP were quantified using ImageJ. A ROI corresponding to this time window was manually selected in ImageJ, and the fluorescence intensity was extracted using the “Plot Profile” function. Intensity trajectories were plotted and analyzed using Origin. Subsequently, kinetic function fitting was performed on the fluorescence intensity-time plots using Origin software (OriginLab), yielding the time to half-maximal fluorescence intensity (T_1/2_) at different EXD2-eGFP concentrations.

#### Hill equation fitting

To quantitatively analyze the binding characteristics of EXD2 to RDHs, we performed fitting analysis using the Hill curve. By calculating the average fluorescence intensity of EXD2 on RDHs within a time window of 10 to 40 s, we could effectively reduce the impact of two types of potential interfering factors: the sharp decrease in EXD2 signals caused by transient binding events, and the gradual signal attenuation induced by photobleaching (Supplementary Fig. S14). Using the average intensity value at this equilibrium state as the dependent variable (*Y*-axis) and the corresponding protein concentration as the independent variable (*X*-axis), we employed the Hill equation to describe the cooperative binding behavior, with its expression shown below:


\begin{eqnarray*}
y = {{V}_{\rm max}}\frac{{{{{\left[ {EXD2} \right]}}^n}}}{{{{k}^n} + {{x}^n}}},
\end{eqnarray*}

where *y* represents the average fluorescence intensity, *V*_max_ is the maximum intensity value at saturation, *k* denotes the apparent half-saturation concentration, and *n* stands for the Hill coefficient.

#### Force–extension relationship and model fitting of a single RDH

The mechanical properties of single 12.3-kbp RDH and 12.3-knt ssDNA molecules were characterized using a dual-trap optical tweezers system. Individual nucleic acid molecules were tethered between two streptavidin-coated polystyrene microspheres via biotin–streptavidin linkages. Force–extension relationships were determined by stretching the tethers at a constant rate of 0.2 μm/s until the tension reached 30 pN. To facilitate direct comparison between the mechanical responses of the two different nucleic acid states, the measured extension was normalized by dividing the total distance by 12.3k base pairs or nucleotides. The resulting curves represent the extension per unit length expressed in units of nm/bp or nm/nt. Experimental force–extension data for RDH were analyzed using the modified worm-like chain (mWLC) model [39, 40]:


\begin{eqnarray*}
\frac{{\mathrm{x}}}{{{{{\mathrm{L}}}_0}}} = 1 - \frac{1}{2}{{\left( {\frac{{{{{\mathrm{k}}}_{\mathrm{B}}}{\mathrm{T}}}}{{{\mathrm{FP}}}}} \right)}^{1/2}} + \frac{{\mathrm{F}}}{{{{{\mathrm{K}}}_{\mathrm{s}}}}},
\end{eqnarray*}

where *F* represents the tensile force applied to both ends of the nucleic acid strand, *x* denotes the extension of the strand under force *F, L_0_* is the contour length of the molecule, *P* is the persistence length (typically 56 nm), *K_s_* represents the elastic stretch modulus (fixed at 1500 pN), and *k_B_* is the Boltzmann constant. The ssDNA stretching data were fitted with a discrete elasticity model to extract the respective physical parameters.

#### Calculation of the number and rate of nucleic acids cleaved by EXD2

The number of cleaved ribonucleotides during RDH degradation was quantified by analyzing the force-dependent extension of the nucleic acid tether. According to the mWLC model for RDHs and the freely jointed chain (FJC) model for ssDNA [41], the conversion of the double-stranded hybrid into ssDNA produces a predictable change in molecular extensibility at a constant force. The total number of nucleotides cleaved (*N*) was calculated using the formula:


\begin{eqnarray*}
N = \frac{{\Delta L}}{{{{d}_{\mathrm{ssDNA}}}\left( F \right) - {{d}_{\rm RDH}}\left( F \right)}},
\end{eqnarray*}

where *ΔL* represents the observed change in tether extension at the applied force *F*. The terms *d*_ssDNA_(*F*) and *d*_RDH_(*F*) denote the extension per nucleotide of ssDNA and the extension per base pair of RDH at force *F*, respectively.

### Cell culture, transfection, and immunofluorescence

HeLa cells were cultured at 37°C under standard conditions (humidified atmosphere, 5% CO_2_) in Dulbecco’s modified Eagle’s medium supplemented with 10% fetal bovine serum (Gibco) and 1% penicillin–streptomycin. For transfection, the relevant plasmid DNA was transfected using LipoMax™ reagent (Sudgen, 32012).

For immunofluorescence, HeLa cells were seeded on coverslips overnight and then fixed with 4% paraformaldehyde for 10 min. After fixation, cells were washed three times with PBS and blocked with 5% BSA in PBS for 1 h. Cells were subsequently incubated with the indicated primary antibodies, followed by incubation with Alexa Fluor-488, -568, or -647 conjugated secondary antibodies (1:1000 dilution). Nuclei were stained with DAPI for 3 min. Finally, coverslips were mounted on glass slides and imaged using a Nikon AXR microscope (Tokyo, Japan). Super-resolution imaging was performed using a Multimodality Structured Illumination Microscopy (Multi-SIM) system equipped with a 100×/1.49 NA oil objective (Nikon CFI SR HP Apo), solid-state lasers (561 nm, 640 nm), and an sCMOS camera (Photometrics Kinetix). Serial Z-stacks were acquired in Stacked Slices-SIM mode. SIM image stacks were reconstructed using SI-Recon 2.23.3 (NanoInsights) with the following parameters: pixel size of 30.6 nm, channel-specific optical transfer functions, a Wiener filter constant of 0.01 for 2D mode, and removal of negative intensity backgrounds. Reconstructed images were further denoised by applying a total variation constraint.

### Immunoblotting

Cells were lysed in cold RIPA buffer (Beyotime) supplemented with 1 mM phenylmethylsulfonyl fluoride and a protein inhibitor cocktail (Roche Diagnostics, 04 693 132 001) for 30 min on ice. Lysates were centrifuged at 12 000 × rpm for 10 min, and protein concentrations were determined using the Bio-Rad protein assay. Approximately 25 µg of protein lysate per sample was separated by SDS–PAGE under reducing conditions and transferred onto nitrocellulose membranes. After incubation with primary antibodies, membranes were probed with fluorescent dye-labeled secondary antibodies (Invitrogen) and scanned using an Odyssey infrared imager (9120, LI-COR Biosciences, Lincoln, NE).

### S9.6 dot blot

Genomic DNA was extracted from HeLa cells using the DNA extraction kit (Vazyme), adding RNasin ribonuclease inhibitor (Promega) for protection against RNases. The extracted DNA samples were spotted onto a positively charged nylon membrane. Then, crosslink nucleic acid to the membrane with UV light: 120 mJ/cm^2^ at 254 nM. After blocking the membranes with 5% milk in 1× PBS, membranes were incubated overnight with the anti-S9.6 antibody. Following primary antibody incubation, the membrane was incubated with peroxidase-conjugated secondary antibody. The dot signals were visualized using the Touch Imager Pro Imaging System (e-BLOT Life Science, China). After visualization, the membranes were stained with methylene blue to confirm sample loading. Dot blot intensities were quantified using ImageJ software.

### Statistical analysis

To determine the appropriate statistical approach, data were first subjected to normality testing using the Shapiro–Wilk test. For datasets that conformed to a normal distribution, one-way analysis of variance (ANOVA) was subsequently performed to assess the statistical significance between groups. All analyses were conducted using Origin 2023.

## Results

### Single-molecule analysis unveils EXD2 cooperative binding to RDHs

**Figure 1.**
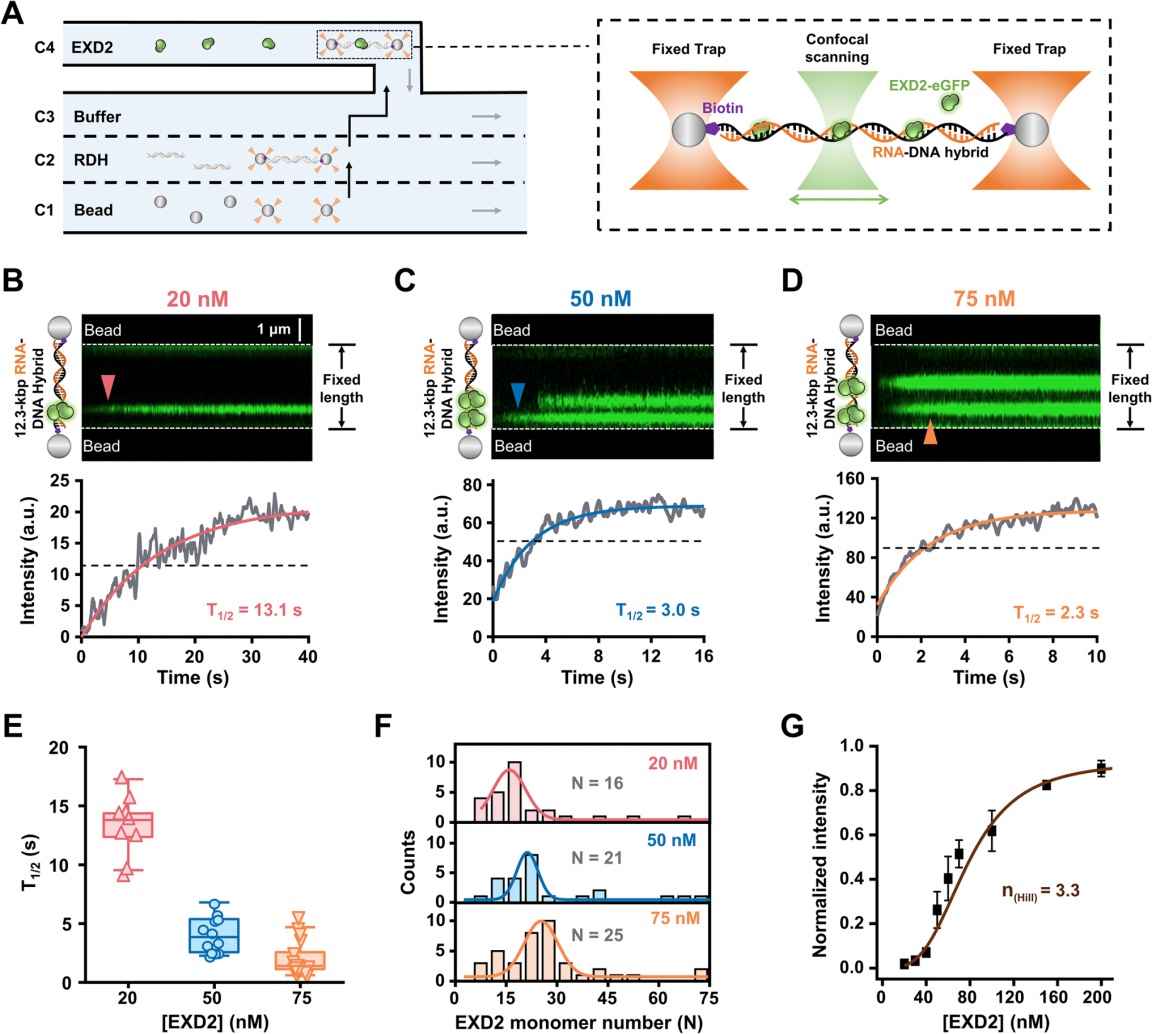
Single-molecule analysis unveils cooperative EXD2 binding to RDHs. (**A**) Overview of the single-molecule experimental setup. Black arrows indicate the experimental workflow, and gray arrows denote the direction of fluid flow. The right panel shows a zoomed-in view. A biotinylated RDH template is suspended between two streptavidin-coated beads manipulated by two optical traps. Meanwhile, confocal lasers repeatedly scanned along the template. (**B–D**) Upper panel: Representative time-lapse fluorescence trajectories of bare RDHs sequentially transferred into channels containing EXD2-eGFP at the indicated concentrations. Black segments indicate bead boundaries. Scale bar: 1 μm. Lower panel: Corresponding fluorescence intensity dynamics of EXD2 multimers on RDHs. T1/2 denotes the time required to reach half-maximal fluorescence intensity. Colored arrows designate distinct binding events. (**E**) Time to half-maximal fluorescence intensity (T_1/2_) of EXD2 multimers at indicated concentrations. The data are shown in mean ± SD. *n* = 9, 12, and 18. (**F**) Gaussian-fitted distributions of EXD2-eGFP multimer numbers at indicated concentrations. *N* indicates the number of analyzed multimers. (**G**) Quantification of total EXD2-eGFP fluorescence intensity on RDHs as a function of EXD2 concentration. Data are presented as mean ± SEM. The brown curve represents Hill equation fitting.

To comprehensively understand how EXD2 engages and processes a long stretch of an RDH molecule, we employed an optical tweezers-based fluorescence imaging approach and monitored their dynamic interplay at the single-molecule level (Fig. 1A). To this aim, we purified enhanced green fluorescent protein-tagged EXD2 (EXD2-eGFP) and synthesized a 12.3-kbp RDH fragment with biotin modifications at both DNA ends (Supplementary Fig. S1) [35]. Using a multi-channel flowcell and dual optical traps, we suspended a single RDH molecule between two streptavidin-coated microspheres (Fig. 1A). After verifying a single RDH molecule by its intrinsic force–extension behavior [35, 42, 43], the tether was swiftly transferred into a protein-containing channel and held at a constant length of 3.6 μm (~10 pN applied on the tether) (Fig. 1A). In this channel, we used confocal line scanning to generate kymographs that can exhibit real-time fluorescence signals of EXD2-eGFP bound to RDH (Fig. 1) [37].

After verifying that the fluorescently labeled EXD2 retained similar RDH binding and cleavage activities (Supplementary Fig. S2), we started the single-molecule assay with a low EXD2-eGFP concentration of 1, 5, and 10 nM. However, we barely detected its binding events on RDHs under these conditions (Supplementary Fig. S3). Upon elevating the EXD2 concentration to 20 nM, we immediately noticed EXD2-eGFP binding to RDH (Fig. 1B). To our surprise, following the appearance of the fluorescence signals of EXD2-eGFP, it continued to expand and, as reflected by the time to half-maximal fluorescence intensity (T_1/2_), reached equilibrium after tens of seconds (Fig. 1B and E). To examine whether these observations are protein concentration-dependent, we performed the experiments in the presence of 50 and 75 nM EXD2, respectively. We found that the binding events increased along with the elevation in the EXD2 concentration (Fig. 1C–E). Additionally, the times required for the binding events to reach equilibrium were significantly shortened to a few seconds. Under a high protein concentration of 75 nM, it is also noticeable that pre-assembled EXD2 multimers directly docked onto the RDH, indicating that EXD2 is capable of self-multimerization in solution (Fig. 1D and Supplementary Fig. S4). To determine the protein numbers within each steady EXD2 cluster, we compared their fluorescence intensities with that of a single dCas9-eGFP/sgRNA complex (Supplementary Fig. S5A). Each cluster contained tens of EXD2 molecules, with the number slightly increasing with the protein concentration (Fig. 1F and Supplementary Fig. S5B).

The continuous expansion of the fluorescence signals suggests that EXD2 may bind to RDHs in a cooperative manner, wherein, post the initial binding, additional EXD2 proteins in solution were quickly recruited to form stable protein clusters on RDHs. To further verify that, we conducted experiments across a wide range of EXD2-eGFP concentrations and analyzed the total fluorescence signals of EXD2-eGFP bound to RDHs after 30 s. In alignment with the cooperative binding mechanism, steady-state binding curves exhibited a sigmoidal shape, and Hill-equation fitting yielded a coefficient of 3.3 (Fig. 1G).

In conclusion, EXD2 proteins cooperatively bind to RDHs, which creates high-affinity assemblies on RDHs. These results reveal a fundamental mechanism underlining the EXD2–RDH interplay.

### EXD2 is a slow and non-processive exonuclease toward tensioned RDHs

**Figure 2.**
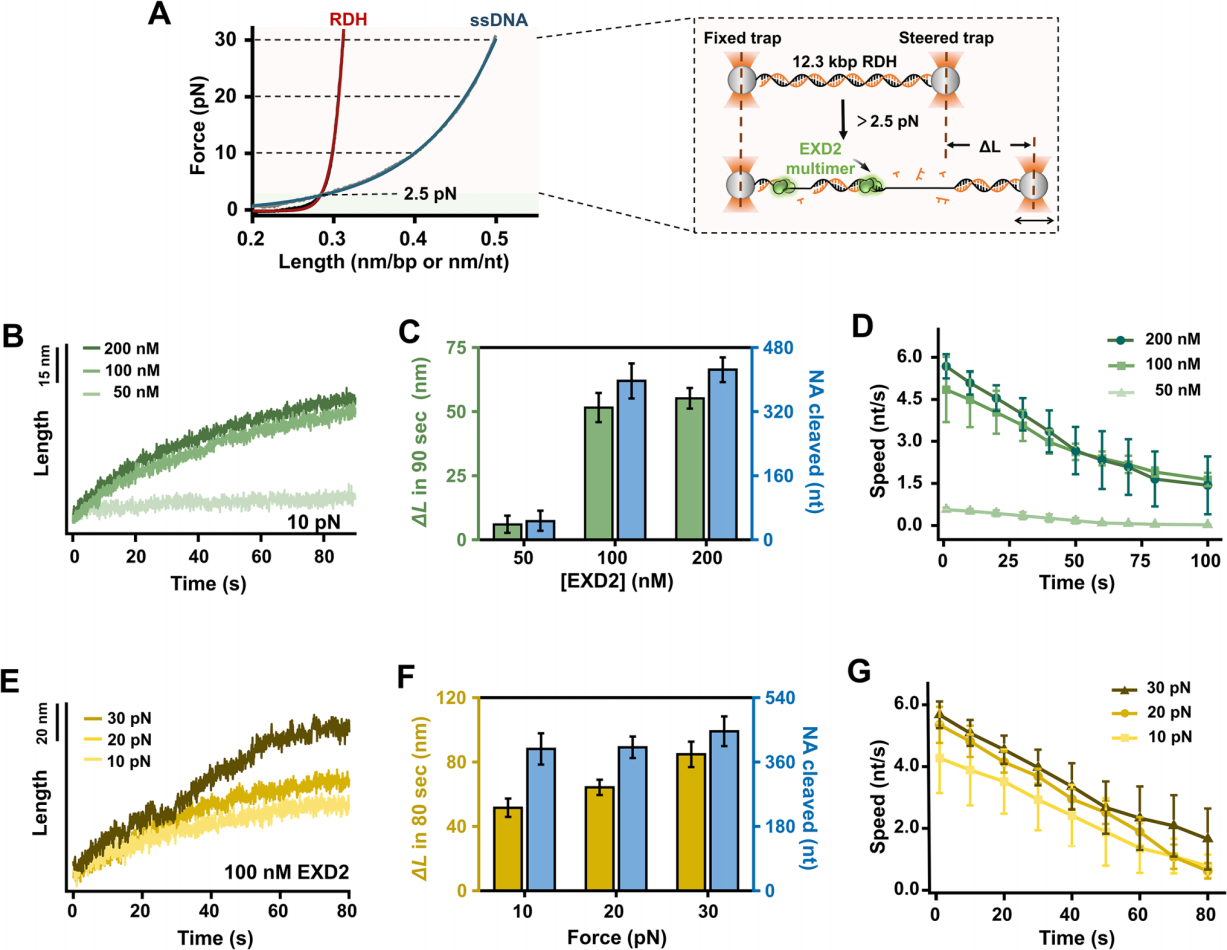
EXD2 slowly digests RDHs under a high force. (**A**) Predicted force–extension behaviors of a 12.3 kbp RDH (red) and ssDNA (blue), modeled using the mWLC and FJC models, respectively. Below ~2.5 pN, enzymatic conversion of RDH into ssDNA is expected to result in an apparent shortening of the molecular extension. The panel on the right shows a schematic of the dual-trap optical tweezers assay used to monitor EXD2-mediated processing of single RDH molecules. (**B**) Representative extension-time trajectories of single RDH molecules under a constant tension of 10 pN, treated with EXD2 at indicated concentrations. (**C**) Quantification of extension changes (*ΔL*) and the corresponding number of nucleotides processed for different EXD2 concentrations at 10 pN. Data represent mean ± SD from multiple single-molecule trajectories. *n* = 11. (**D**) Instantaneous reaction rates extracted from single-molecule trajectories under 50, 100, and 200 nM EXD2 at 10 pN tension, with measurements taken every 10 s. (**E**) Time traces showing RDH extension changes at elevated forces (≥10 pN) in the presence of 100 nM EXD2. (**F**) Summary of *ΔL* and calculated nucleotide processing under high-force conditions (≥10 pN) at a fixed EXD2 concentration (100 nM). Bars represent mean ± SD. *n* = 7. (**G**) Instantaneous reaction rates extracted from single-molecule trajectories at different applied forces (≥10 pN) in the presence of 100 nM EXD2.

After unveiling the cooperative binding mechanism, we next asked how EXD2 dynamically processes RDHs. Given that EXD2 prefers to digest RNA within the RDHs [28, 33], it is expected that EXD2-catalyzed cleavage would mainly convert RDHs into ssDNA. Thus, we exploited the distinct mechanical properties of RDH and ssDNA to monitor this process in real time. To this end, we first determined the force–extension relationships of a single RDH and ssDNA molecule. Based on the obtained nucleic acid stretching curves, the ssDNA length is expectedly longer than that of the RDH curves at forces above the crossover point of 2.5 pN and shorter at forces below (Fig. 2A), consistent with previous studies [35].

Using a high-frequency feedback loop, we applied a constant force of 10 pN on the 12.3-kbp RDH while recording its length change in the channel containing 50 nM EXD2 [35]. Previous studies have demonstrated that Mn^2+^ serves as the most potent cofactor that maximizes EXD2’s *in vitro* catalytic cleavage efficiency [19, 33]. Therefore, Mn^2+^ was utilized in the single-molecule digestion assays to achieve robust and detectable degradation rates under mechanical tension. As expected, the tether length indeed increased, albeit at extremely low rates with minimal processivities (Fig. 2B and C). In contrast, depletion of Mn^2+^ in the reaction system abolished the length increase, supporting that the detected tether extension is a result of catalytic digestion of RDH by EXD2 (Supplementary Fig. S6). According to the force–extension relationships, the hybrid length extended by ~6 nm over 90 s, corresponding to cleavage of only ~46 nucleotides (Fig. 2B and C). Increasing EXD2 concentration to 100 and 200 nM substantially enhanced degradation activity, and yet EXD2 remained intrinsically slow (4 and 5 nt/s) and non-processive (Fig. 2B and C). Instantaneous velocity analysis further revealed a gradual decline in cleavage rate over time, suggesting limited catalytic persistence (Fig. 2D). In comparison, the catalytic activities of EXD2 are several orders of magnitude weaker than specialized processing enzymes that degrade RNA in RDHs, such as RNase H [44, 45]. To verify that the apparent length increases were mainly caused by EXD2-mediated RNA degradation, we combined optical tweezers with confocal fluorescence imaging. A 12.3-kbp hybrid containing Cy3-UTP-labeled RNA was stretched at 10 pN and transferred into a channel containing 200 nM EXD2 (Supplementary Fig. S7A). As the tether slowly elongated, the fluorescent RNA signal gradually disappeared, indicating progressive loss of Cy3-labeled RNA from the hybrid (Supplementary Fig. S7A). These results identify EXD2 as a slow and poorly processive exonuclease on long RDH substrates.

Our previous study has demonstrated that RDH unwinding is a prerequisite for EXD2 to access and cleave RNA [33], which raises the possibility that increased mechanical tension might promote EXD2’s nuclease activity by facilitating hybrid destabilization. To test this hypothesis, we elevated the force applied to the RDH to 20 pN and quantified the resulting digestion kinetics at 100 nM EXD2. Quantitative analysis revealed that the number of ribonucleotides cleaved by EXD2 at 20 pN increased by ~25% compared to that at 10 pN (Fig. 2E and F). Further elevating the tension to 30 pN resulted in a similarly modest increase (Fig. 2E and F). Notably, instantaneous velocity analysis supports that these higher tensions still failed to significantly improve the catalytic persistence of the enzyme (Fig 2G). Thereby, EXD2’s nuclease activity is only modestly sensitive to increased mechanical load.

In summary, our findings establish that EXD2 is a slow, non-processive exonuclease toward tensioned RDHs and that its intrinsic nuclease activity displays weak sensitivity to elevated substrate tension. These properties distinguish EXD2 from robust RDH-processing nucleases and suggest that EXD2 may fulfill a specialized, context-dependent role in hybrid metabolism rather than functioning as a general, high-efficiency degradative enzyme.

### EXD2 under high concentrations dominantly condenses relaxed RDHs

**Figure 3.**
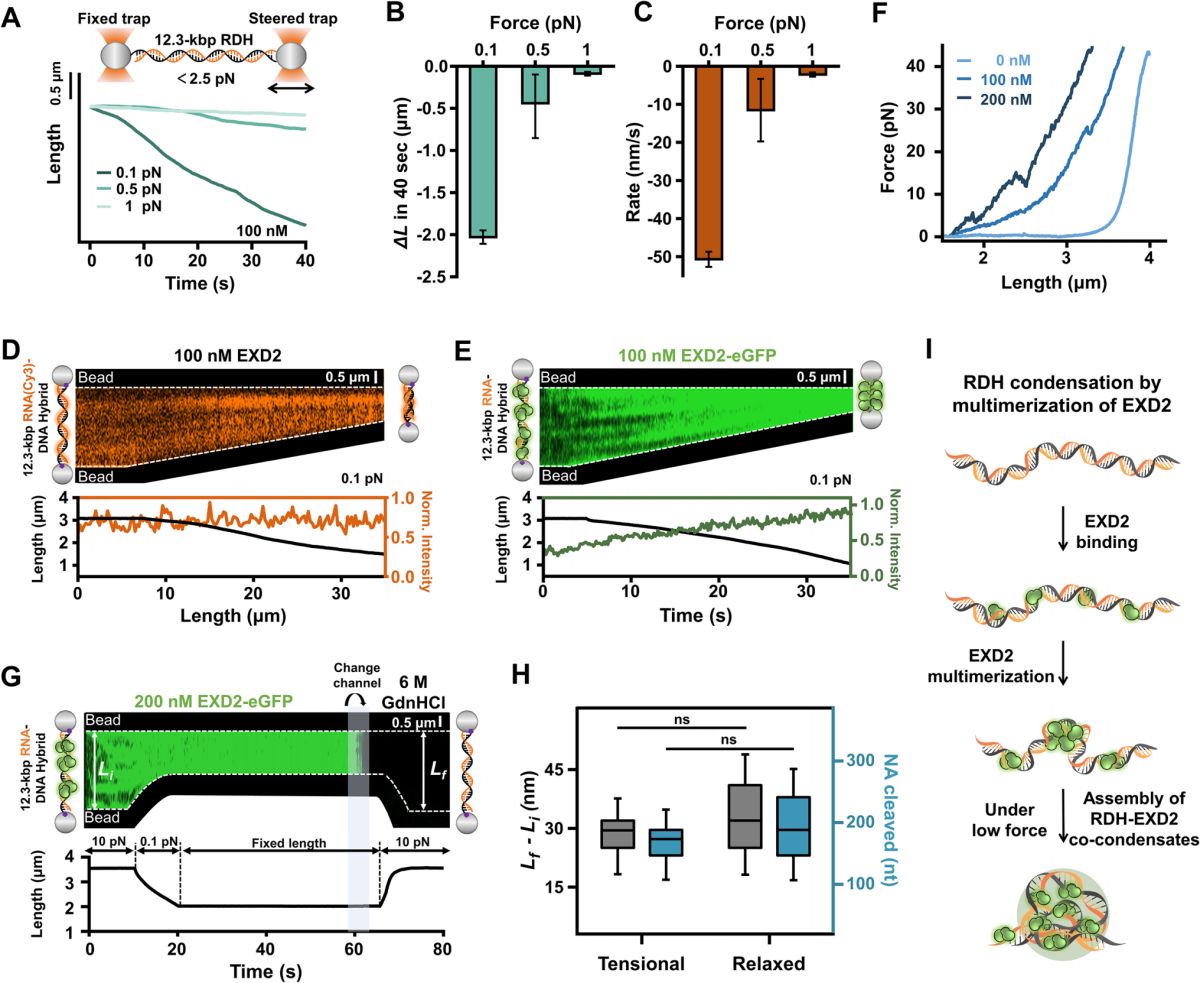
EXD2 multimerization induces RDH condensation against a low force. (**A**) RDH shortening under low mechanical tension. Top, schematic illustrating RDH compaction when the applied force is below ~2.5 pN. Bottom, representative extension-time traces of a 12.3-kbp RDH in the presence of 100 nM EXD2 at forces lower than 2.5 pN. (**B**) Quantification of RDH shortening. Mean change in tether length (*ΔL*) measured within 40 s at different low-force conditions with 100 nM EXD2. Bars represent mean ± SEM. (**C**) Mean shortening rates measured within 40 s under different low-tension conditions in the presence of 100 nM EXD2. Bars represent mean ± SEM. (**D**) RDH compaction occurs without RNA loss. Top, confocal kymograph of Cy3-labeled RNA within an RDH at 0.1 pN in the presence of 100 nM EXD2. Bottom, time traces of RDH length (black) and total Cy3 fluorescence intensity (orange). (**E**) EXD2 accumulation correlates with RDH compaction. Top, confocal kymograph of 100 nM EXD2-eGFP binding to a single RDH at 0.1 pN. Bottom, corresponding time traces of RDH length (black) and total EXD2-eGFP fluorescence intensity (green). (**F**) Mechanical stability of EXD2–RDH condensates. Force–extension curves of naked RDHs and RDHs incubated with 100 or 200 nM EXD2 (blue). (**G**) Experimental scheme to test the effect of condensation on EXD2 nuclease activity. An RDH was first compacted by 200 nM EXD2 at 0.1 pN and then transferred to 6 M guanidinium hydrochloride to dissociate EXD2. *L_i_* and *L_f_* denote RDH lengths measured at 10 pN before and after EXD2 removal, respectively. Shaded regions indicate channel exchange. (**H**) Quantitative analysis of the values of *L_f_*–*L_i_* and the corresponding nucleotide equivalents. The reaction with 200 nM EXD2 for 60 s under a constant force of 10 pN (gray), and the co-reaction for 60 s after compaction at 0.1 pN (blue). (**I**) Model of force-dependent EXD2 function on RDHs.

Next, we examined how EXD2 behaves on relatively relaxed RDHs, which are more representative of *in vivo* nucleic-acid states. To test that, we revisited the RDH cleavage assays under a low force of 1 pN (Fig. 3A). Indeed, in the 100 nM EXD2 channel, the RDH tether length was shortened for ~87.7 nm within 40 s, yielding a shortening rate of around 2.2 nm/s (Fig. 3A–C). Moreover, further reducing the applied force to 0.5 and 0.1 pN led to a dramatic acceleration of RDH shortening (Fig. 3A–C).

Next, we aimed to explore the molecular mechanism governing EXD2-induced RDH shortening. As aforementioned, the conversion of RDH to ssDNA by EXD2 is supposed to cause a decrease in the tether length under a force lower than 2.5 pN (Fig. 2A). However, based on the force–extension relationships, we estimated a cleavage rate of 50.7 nm/s (~461 nt/s) under 0.1 pN, which is several orders of magnitude higher than the canonical degradation rate of EXD2 (Fig. 3B and C). In addition, depletion of Mn^2+^ cannot completely abolish the shortening of RDHs under this condition (Supplementary Fig. S8). This striking discrepancy indicates that the observed length reduction may not be due to enzymatic degradation alone. To further validate that, we used a 12.3-kbp RDH containing Cy3-labeled RNA by incorporating Cy3-UTP during transcription, enabling real-time monitoring of RNA integrity. Under 100 nM EXD2 and 0.1 pN force, the hybrid gradually shortened with the overall fluorescence intensity of the RNA-Cy3 largely maintained (Fig. 3D). Thereby, the detected shortening does not completely originate from the EXD2 cleavage. Notably, along with the shortening, bright punctate fluorescence emerged randomly along the RDH, suggesting that the RDH was locally condensed. Considering the strong cooperative multimerization of EXD2 on RDHs (Fig. 1), we hypothesized that the shortening observed at low force arises from EXD2–RDH co-condensation. To examine that, we used EXD2-eGFP to monitor its status during RDH condensation. As expected, in the presence of 100 nM EXD2-eGFP, RDH was robustly condensed under 0.1 pN (Fig. 3E). During this process, EXD2-eGFP discretely accumulated into multimers along the hybrid in parallel with progressive tether shortening. Notably, the fluorescence intensity of EXD2-eGFP clusters scaled linearly with the degree of RDH compaction, supporting a model in which EXD2 multimerization actively promotes and stabilizes RDH condensation (Fig. 3E). These observations confirm that RDH shortening reflects local, stochastic co-condensation between EXD2 and the hybrid, rather than enzymatic cleavage.

Next, we examined the mechanical stability of these condensates by stretching EXD2-condensed RDH tethers. In this assay, after confirming multimeric EXD2 binding, we reduced the tether length to ~1.5 μm to relax the RDH (close to 0 pN) for 5 s, followed by re-stretching it at a constant speed of 0.1 μm/s. Force–extension analysis revealed that even up to ~40 pN, EXD2-bound RDHs exhibited a markedly reduced contour length compared with naked RDHs (Fig. 3F). Throughout re-stretching, EXD2 fluorescence puncta remained stable, and the multimers persisted as dynamically arrested micro-condensates (Supplementary Fig. S9). These results demonstrate that preassembled EXD2 multimers compress relaxed RDHs and form mechanically robust condensates capable of withstanding tens of piconewtons of force (Fig. 3F).

We then asked whether EXD2-mediated condensation influences its nuclease activity toward RDHs. To address this, we measured the tether length change before and after partial RDH condensation. In detail, a single 12.3-kbp RDH was first incubated with 200 nM EXD2-eGFP at an initial length of ~3.6 μm (*L_i_*, corresponding to 10 pN) for ~10 s to allow protein binding, then adjusted to ~2 μm (0.1 pN) for RDH condensation in 60 s (Fig. 3G). To measure the final tether length (*L_f_*), the tether was subsequently transferred to a denaturant (guanidine hydrochloride, GdhHCl)-containing buffer to dissociate RDH-bound EXD2 and maintained a force of 10 pN (Fig. 3G). Control experiments confirmed that the denaturant did not affect the mechanical properties of the examined nucleic acids (Supplementary Fig. S10). Comparison of tether length changes revealed no significant difference between tensioned and relaxed RDHs, indicating that EXD2-mediated condensation does not apparently alter its nuclease activity (Fig. 3H).

Altogether, these results reveal a force-dependent promiscuous behavior of EXD2 on RDHs: Under high tension, EXD2 acts as a slow and poorly processive exonuclease, whereas under low tension it undergoes cooperative oligomerization and co-condensation with RDHs to form mechanically stable nucleoprotein assemblies (Fig. 3H). Importantly, this condensation state does not apparently affect EXD2’s nuclease activity, suggesting that EXD2 switches from a degradative to a structural mode depending on the mechanical state of its substrate.

### EXD2-induced RDH condensation protects against rapid degradation

**Figure 4.**
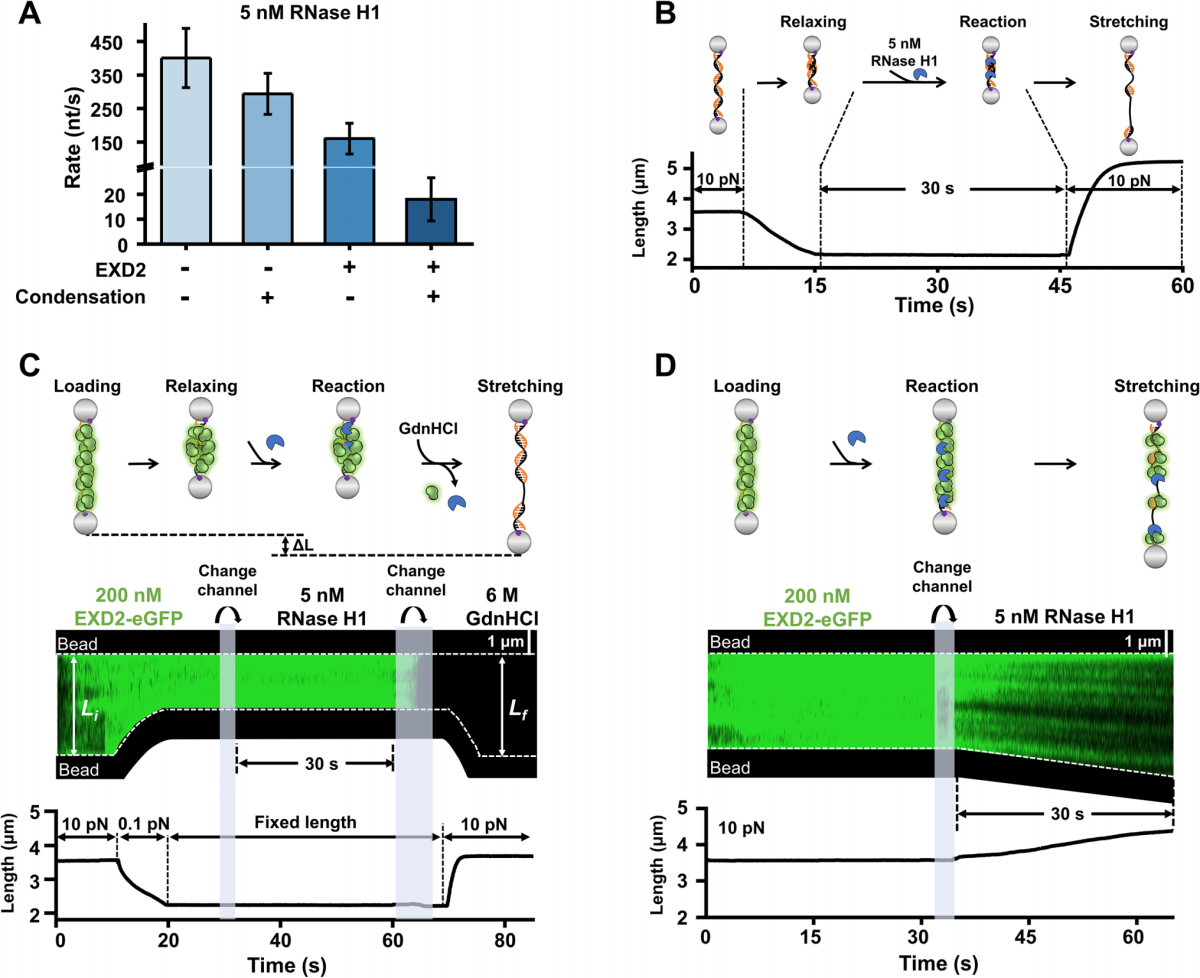
EXD2-mediated RDH condensation protects against degradation. (**A**) Quantification of the rate (*ΔL/Δt*) after 30 s of RNase H1 treatment for naked RDHs, EXD2–RDH co-condensates, and EXD2-bound but non-condensed RDHs. (**B**) The naked RDH, stretched at 10 pN, is relaxed to a length of 2 μm, then transferred to a channel containing 5 nM RNase H1 for a 30-s reaction, and finally re-stretched at 10 pN. (**C**) Top: Cartoons illustrating the experimental procedures. Middle: Real-time fluorescence line scans showing EXD2-eGFP bound to and condensed on the RDH. Bottom: Corresponding bead–bead distance and applied force as functions of time. (**D**) Control workflow in which an RDH tether was incubated with EXD2 at 10 pN without inducing condensation, followed by direct transfer into the RNase H1 channel for a 30-s reaction.

Since single-molecule analysis has demonstrated robust RDH condensation by EXD2, it is tempting for us to speculate that EXD2–RDH co-condensates may protect RDHs from enzymatic degradation. We employed RNase H1, a nuclease that specifically recognizes and rapidly cleaves the RNA strand within RDHs in a Mg^2+^-dependent manner [35], to test this hypothesis. We first measured RNase H1’s cleavage rate at the single-molecule level. In alignment with our previous findings [35], naked RDHs at 10 pN underwent rapid degradation when exposed to 5 nM RNase H1, generating ssDNA at a rate of ~401 nt/s (Fig. 4A and Supplementary Fig. S11). Similarly, relaxed RDHs (~0.1 pN) were also efficiently degraded by RNase H1 (Fig. 4B).

We next asked whether EXD2-condensed RDHs could resist RNase H1-mediated digestion. To this end, we incubated a single RDH molecule with 200 nM EXD2 under a low force of ~0.1 pN until the tether was compacted to ~2 μm (Fig. 4C). This EXD2-condensated RDH tether was then transferred into a channel containing 5 nM RNase H1 for 30 s. Subsequently, the tethers were moved into a GdhHCl channel to dissociate EXD2 and RNase H1 from RDH [46, 47]. Finally, the RDHs were stretched at 10 pN to assess the extent of degradation based on the final tether length (Fig. 4C). We found that the rate of the tether length change (*ΔL/Δt*) detected was markedly lower than that of naked RDHs (Fig. 4A–C), indicating that condensation substantially suppresses RNase H1 activity.

To exclude the possibility that this effect simply arises from EXD2 preventing RNase H1 from accessing the hybrid, we performed a control experiment in which RDHs were pre-coated with EXD2 at 10 pN without inducing condensation, and then transferred into the RNase H1 channel for 30 s (Fig. 4D). Under these conditions, the degradation rate was reduced by approximately half relative to naked RDHs, but remained an order of magnitude greater than that observed for condensed substrates (Fig. 4B). Therefore, EXD2–RDH co-condensation, rather than mere protein occupancy, is required for robust protection against RNase H1.

Taken together, these findings establish that EXD2-mediated condensation generates a protective nucleoprotein assembly that strongly resists degradation by other nucleases. The protective effect of condensation likely arises from the tight and stable association between EXD2 oligomers and RDHs, which may sterically hinder nuclease access or create a dense protein barrier that limits substrate accessibility [38, 48].

### EXD2 recognizes damage sites and initiates multimerization for RDH protection

**Figure 5.**
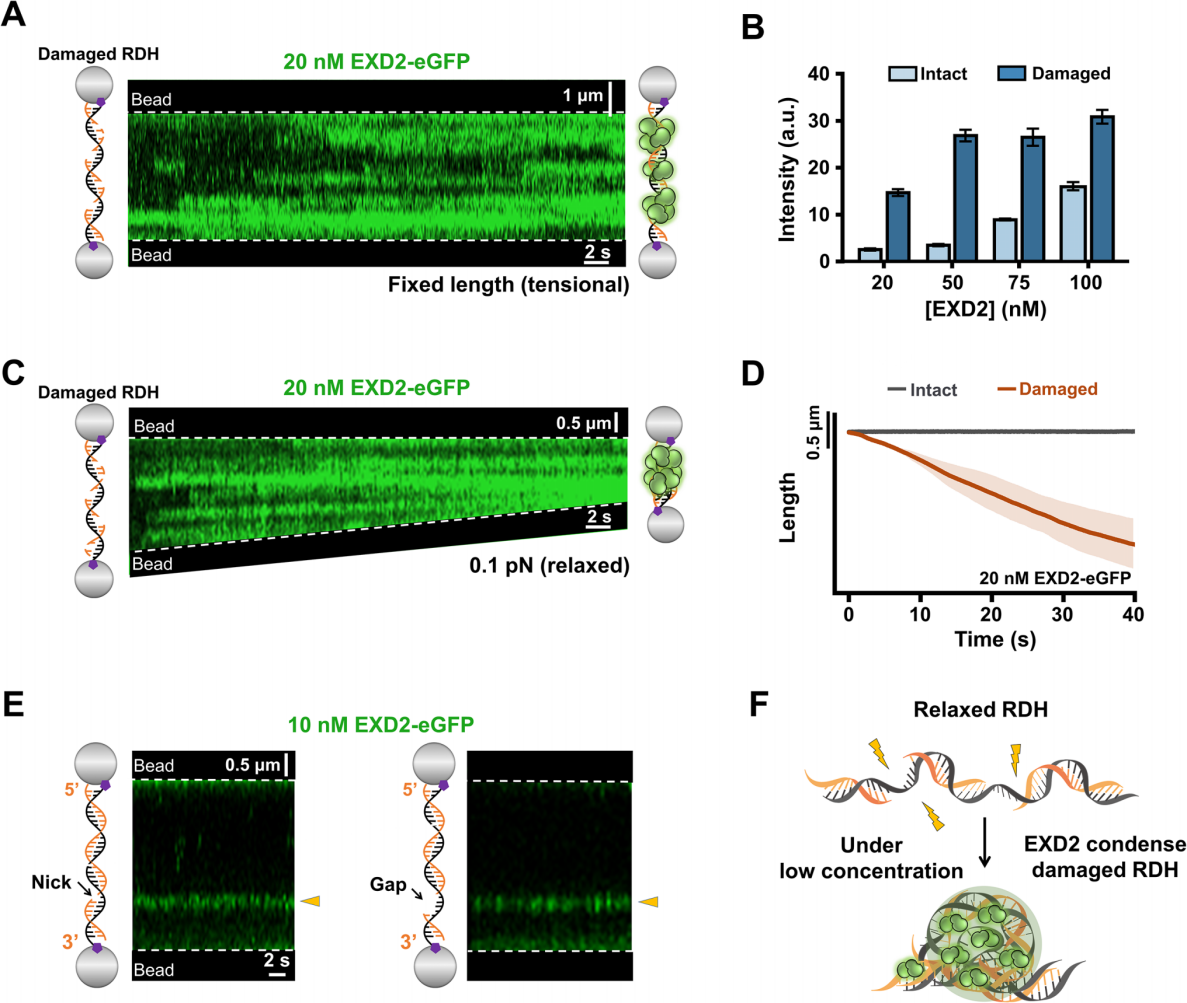
RDH damage promotes EXD2 loading and multimerization for protection. (**A**) Fluorescence line scans of EXD2-eGFP binding to damaged RDHs at fixed tether length. (**B**) Quantification of EXD2-eGFP fluorescence intensity on intact versus damaged RDHs at increasing EXD2 concentrations (20, 50, 75, and 100 nM). (**C**) Real-time fluorescence scans showing EXD2-eGFP binding to RDHs that were briefly treated with 5 nM RNase H1 for 5 s at 0.1 pN to introduce RNA nicks, followed by protein removal with guanidinium hydrochloride and transfer into an EXD2-containing channel under constant low force. (**D**) Statistical analysis of real-time length changes for intact and RNase H1-damaged RDHs upon EXD2 binding under low tension (0.1 pN), showing that RNA damage enables EXD2-driven condensation even at low protein concentrations. *n* = 5. (**E**) Representative fluorescence images showing binding of 10 nM EXD2-eGFP to a nicked RDH (left) and to an RDH containing a 100-nt gap (right). (**F**) Schematic of damaged RDHs condensed by EXD2 at low concentrations.

Previous studies reported the involvement of EXD2 in DNA damage repair [20]. It thus motivated us to ask how EXD2 interacts with damaged RDHs. We then set out to prepare a damaged RDH by treating intact 12.3-kbp RDHs with 5 nM RNase H1 for 5 s to introduce dispersed RNA nicks and gaps on the RDHs. The damaged RDH was then transferred into the 6 M GdhHCl channel to remove RNase H1. Following that, the tether was transferred into a 20 nM EXD2-eGFP channel. To our surprise, compared to the scarce loading on intact RDHs, EXD2-eGFP was observed to immediately accumulate on damaged hybrids (Figs 1B and 5A). Quantitative analysis of the fluorescence intensity over a 40-s observation window revealed a dramatic enhancement of EXD2 binding on damaged RDHs compared with intact substrates (Fig. 5B). We further asked whether damage-enhanced EXD2 loading is sufficient to trigger condensation even at the low protein concentration. Under a low force of 0.1 pN and 20 nM EXD2, progressive shortening of the RDH was observed concomitant with protein accumulation, closely resembling the condensation behavior previously observed at 100 nM EXD2 on undamaged RDHs (Fig. 5C and D).

Our findings raised the possibility that EXD2 can recognize lesion sites within RDHs. In particular, these sites could serve as a molecular signal to promote EXD2 loading and trigger condensation on compromised substrates. To verify that, we engineered a nick-containing RDH substrate in which the RNA strand contained a single discontinuity at one-third of its length, exposing free RNA 3′ and 5′ termini (Supplementary Fig. S12). In a 10 nM EXD2-eGFP channel, fluorescence signals appeared exclusively at the nick position (Fig. 5E). To further approximate the damaged structural context of RDHs, we constructed a hybrid substrate containing a 100-bp ssDNA gap flanked by 4.2-kbp and 8-kbp RNA–DNA duplex regions. On this gapped RDH substrate, EXD2-eGFP at a low concentration of 10 nM again produced discrete binding signals confined to the gap region, confirming that EXD2 preferentially targets damaged or discontinuous sites within RDHs (Fig. 5E). Given that EXD2 is incapable of binding to ssDNA under this condition (Supplementary Fig. S13), these observations support the notion that EXD2 preferentially associates with RNA–DNA junctions or damaged RNA termini within RDHs.

Together, these results demonstrate that damage sites within RDHs act as potent triggers for EXD2 recruitment, cooperative oligomerization, and co-condensation with RDHs (Fig. 5F). By coupling lesion recognition to condensate formation, EXD2 possibly assembles protective nucleoprotein structures at sites of RDH damage, providing a mechanistic link between RNA damage sensing and hybrid stabilization.

### EXD2 dually regulates mitochondrial RDHs in a dose-dependent switching manner

To further validate these molecular mechanisms in cells and bridge the gap between EXD2’s enzymatic versatility and its physiological impact, we sought to address how EXD2 dynamically regulates mitochondrial RDHs *in vivo*. Given the multifaceted roles and dynamic subcellular distribution of EXD2 [19, 22], determining its precise spatial localization is essential for elucidating its regulatory logic toward diverse nucleic acid structures. Although EXD2 is recognized as a key nuclease in resolving hybrids [28], whether it directly accesses the mitochondrial matrix to interact with these substrates and how it maintains spatial specificity on long hybrids has remained poorly understood. To bridge the gap between EXD2’s enzymatic versatility and its physiological impact, we first sought to establish whether the physiological niche of EXD2 overlaps spatially with its RDH substrates.

**Figure 6.**
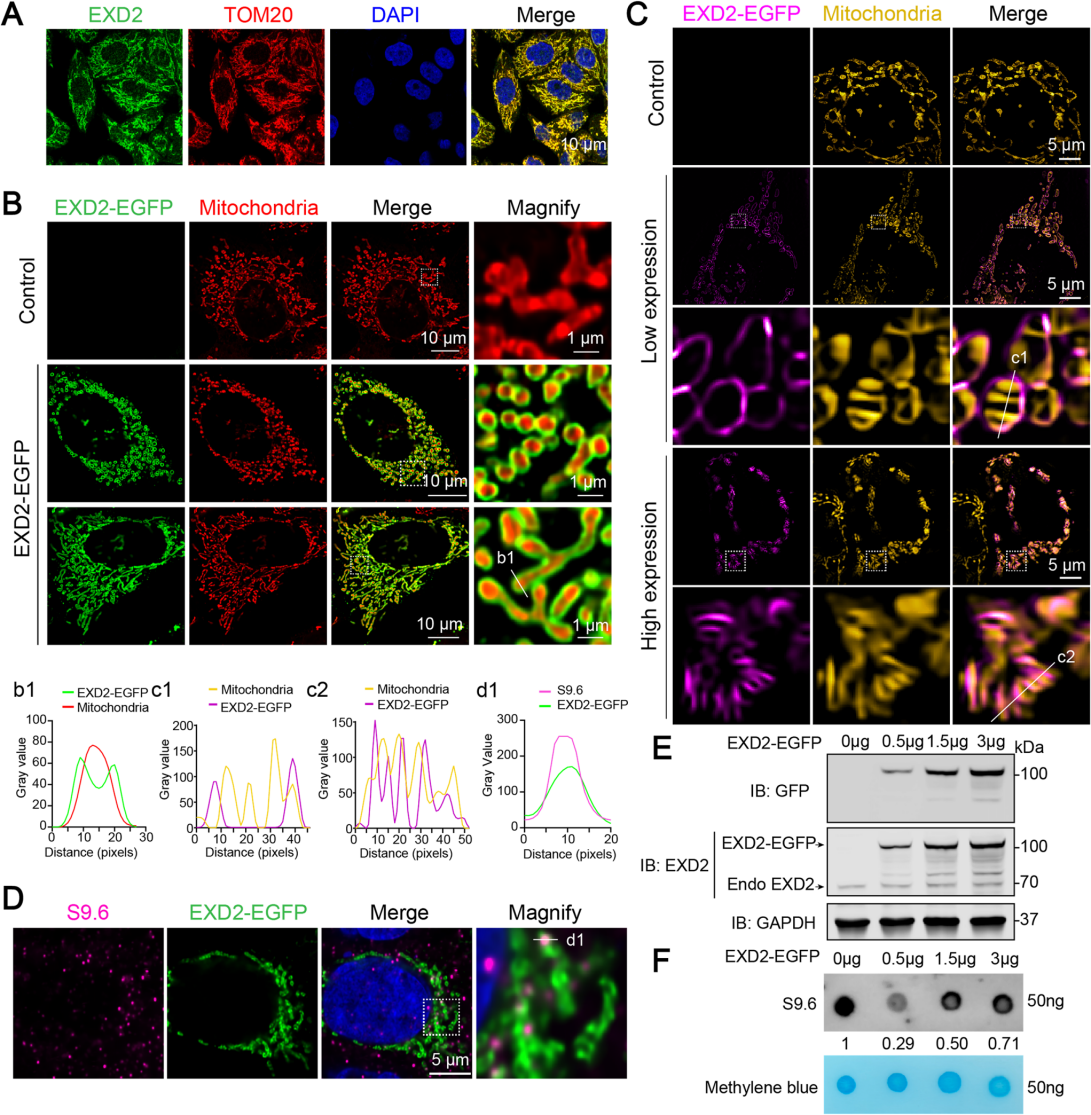
EXD2 dually regulates mitochondrial RDHs in a dose-dependent switching manner. (**A**) Endogenous EXD2 co-localized with OMM marker TOM20. Representative immunofluorescence images of EXD2 (green) and TOM20 (red) in HeLa cells. Nuclei were stained with DAPI (blue). (**B**) Ectopically expressed EXD2-eGFP initially formed ring-like structures surrounding the mitochondria. Representative immunofluorescence images of EXD2 (green) and mitochondria (stained with PK Mito dyes, red) in live HeLa cells. (**C**) Super-resolution microscopic images of EXD2-eGFP and mitochondria (stained with PK Mito dyes) in live HeLa cells. Line-scan analysis (c1, c2, white line) using ImageJ. (**D**) Ectopically expressed EXD2-eGFP co-localized with RDH. Representative immunofluorescence images of EXD2 (green) and S9.6 (magenta) in fixed HeLa cells. Nuclei were stained with DAPI (blue). Line-scan analysis (d1, white line) using ImageJ. (**E**) Immunoblotting analysis of total extracts of HeLa cells transfected with EXD2-eGFP probed for the indicated antibodies. (**F**) Dot blot performed to evaluate the level of RDHs using the S9.6 antibody with 50 ng of genomic DNA. Methylene blue normalization of the S9.6 signal image acquired using ImageJ software.

To determine the steady-state localization of EXD2, we first performed immunofluorescence staining and localized endogenous EXD2 proteins. Consistent with previous reports [19, 21], endogenous EXD2 is predominantly localized to the mitochondria and overlaps with the outer mitochondrial membrane (OMM) marker TOM20 (Fig. 6A and Supplementary Fig. S15). Interestingly, super-resolution imaging revealed that ectopically expressed EXD2-eGFP initially formed ring-like structures surrounding the mitochondria; however, higher expression levels triggered a dynamic redistribution of EXD2 from the OMM to the inner mitochondrial compartment, where it partially co-localized with mitochondrial cristae (Fig. 6B and C). Given that mtDNA is a known hotspot for R-loop/RDH accumulation [49, 50], we examined whether this internal redistribution positioned EXD2 in proximity to mitochondrial RDHs. Our imaging confirmed that exogenous EXD2 indeed co-localizes with these hybrid structures, suggesting that EXD2 is strategically positioned within the mitochondrial compartments where RDH homeostasis is actively maintained (Fig. 6D).

Next, we aimed to assess the impact of EXD2 on global mitochondrial RDH levels. To this end, we performed dot-blot assays using the S9.6 antibody to quantify global hybrid levels. At a low transfection dose of EXD2 (0.5 µg of EXD2 plasmid), we observed a significant reduction in S9.6 signal intensity, confirming that EXD2 acts as a nuclease and promotes RDH degradation under conditions of moderate protein abundance (Fig. 6E and F). Intriguingly, as the transfection dose of EXD2 increased to 1.5 µg and 3 µg, the S9.6 signal showed no further decline, but instead increased substantially relative to the 0.5 µg group (Fig. 6F). Quantitative analysis revealed that the RDH levels in the 1.5 µg and 3 µg groups were approximately two-fold and three-fold higher than those observed in the 0.5 µg group, respectively. These results indicate that EXD2 dually regulates RDH homeostasis in a dose-dependent switching manner. The failure to achieve further degradation at higher concentrations suggests a potential functional switch, wherein EXD2 may transition from a purely degradative nuclease to a distinct mode that stabilizes or shields the hybrids at higher concentrations.

## Discussion

**Figure 7.**
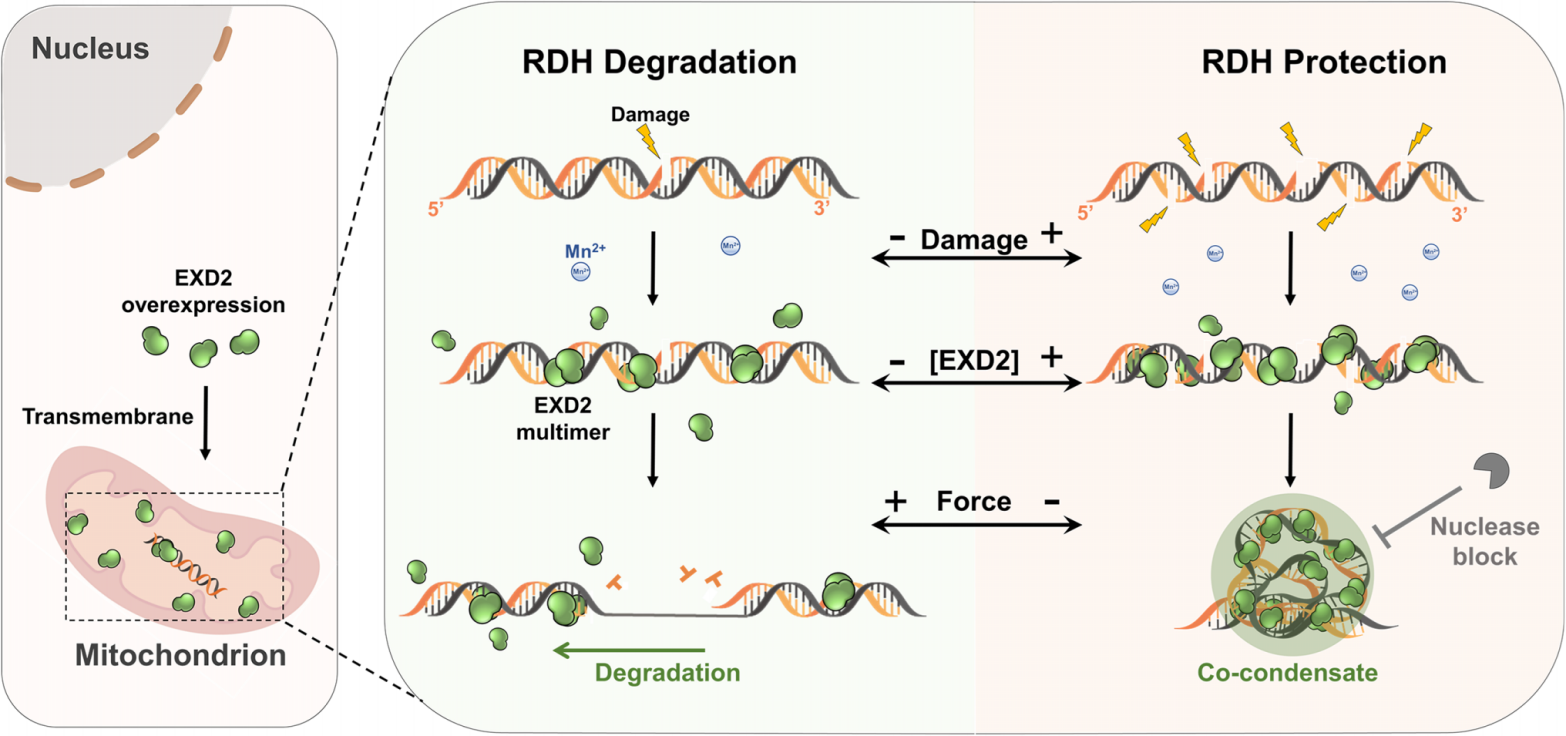
EXD2’s dual and opposing roles in resolving mitochondrial RDHs. Left. EXD2 localizes to mitochondria and targets RDHs upon protein overexpression. Right. EXD2 acts as a promiscuous nuclease with two functional modes regulated by context. RDH Degradation: At moderate damage and high tension, EXD2 degrades the RNA strand, leading to an increase in molecular extension. RDH Protection: Under severe damage, high EXD2 concentration, and low tension, EXD2 undergoes cooperative condensation with RDHs. This forms a micro-condensate that compacts the hybrid and shields it from other nucleases.

In this study, we investigated the dynamic EXD2–RDH interactions and established that EXD2 acts as a promiscuous enzyme regarding the resolution of RDHs [11]. To understand its regulatory logic at the fundamental level, we performed a series of single-molecule experiments with both intact and damaged RDH substrates. As illustrated in Fig. 7, the EXD2 exonuclease cooperatively binds to and slowly digests RDHs; through multimerization, EXD2 can also co-condense RDHs and, conversely, protect them from fast degradation by other nucleases; damage sites within RDHs further stimulate EXD2 binding and protection. Thereby, EXD2 possesses dual and opposing roles in the resolution of RDHs.

A major finding of our work is that EXD2 is a moonlighting enzyme that exhibits both catalytic and non-catalytic activities regarding RDH resolution. Remarkably, EXD2’s functional identity is defined by a conditional bifurcation between degradative and protective modes. As an exonuclease, EXD2 is catalytically competent but intrinsically slow and poorly processive on long RDH substrates. Even under sustained mechanical tension, EXD2 removes RNA only gradually and without persistent directional motion, indicating that its nuclease activity is tightly restrained. Such slow kinetics suggest that EXD2 is not optimized for bulk hybrid clearance, but instead may function in controlled trimming or localized processing of RDHs. On the other hand, a high amount of EXD2 no longer primarily degrades relaxed RDHs but instead undergoes cooperative multimerization and co-condensation with the hybrids. This transition strongly depends on protein concentration and is favored when the substrate is mechanically relaxed (Fig. 7). The resulting EXD2–RDH assemblies are mechanically robust, remaining compacted under forces of tens of piconewtons. Importantly, condensation does not alter EXD2’s intrinsic nuclease activity and yet effectively shields the hybrid from degradation by other nucleases, indicating that oligomerization represents a functional state distinct from catalysis. These observations suggest that EXD2’s activities toward RDHs are dictated by the physical state of the substrate and local protein concentration. This protective activity of EXD2 mirrors the behavior of other key genome maintenance factors, such as the RPA complex, which protects vulnerable ssDNA via co-condensation as well [51, 52]. The involvement of the active condensation of nucleic acids induced by RPA and EXD2 is a unique feature that distinguishes it from the non-compressive status of nucleic acids protected by bound proteins, such as RAD51 on dsDNA [53]. Furthermore, our observation that EXD2 exhibits a preference for structural gaps over simple nicks highlights the role of structural discontinuity at RNA–DNA junctions. Such regions likely present enhanced spatial accessibility and conformational flexibility, lowering the energy barrier for EXD2 docking and subsequent cooperative multimerization. While specialized nucleases are optimized for rapid and exhaustive hybrid clearance [35], EXD2 appears to function as a regulatory gatekeeper that prioritizes substrate stability under low-force regimes. The formation of these dense micro-condensates likely creates a steric barrier that prevents catastrophic degradation during transient periods of mechanical relaxation, such as during the intervals between transcription or replication bursts. By integrating mechanical cues with local protein abundance, EXD2 provides a versatile molecular platform for the high-precision management of mitochondrial RDH homeostasis.

Our study also offers new insights into EXD2 functioning in genome maintenance. RNA damage within RDHs serves as a potent trigger for EXD2 recruitment and oligomerization. Damage sites create high-affinity binding sites that locally concentrate EXD2 and promote multimer formation (Fig. 7). This damage sensitivity provides a plausible mechanism for the selective targeting of vulnerable hybrids. Such recruitment may allow EXD2 to rapidly recognize compromised RDHs and respond by assembling protective condensates at lesion sites. By locally shielding damaged hybrids, EXD2 could prevent excessive or untimely degradation, while preserving the option for subsequent repair or controlled processing. This protective shield is of paramount biological significance to cell survival; while persistent RDHs pose risks to genome integrity, their premature, uncoordinated nucleolytic cleavage under severe cellular stress can be far more destructive. When proper multi-step resolution pathways are overwhelmed, rogue or uncoordinated nucleases can aberrantly incise exposed hybrid domains, rapidly converting transient transcription or replication obstacles into catastrophic DNA DSBs and triggering mitochondrial genome fragmentation [54–56]. Thus, EXD2-mediated co-condensation acts as a critical “kinetic buffer” that temporarily freezes and sequesters compromised substrates, preventing nuclease-driven genome collapse until high-fidelity repair machinery can be safely orchestrated. This behavior aligns with emerging themes in genome maintenance, where damage recognition and structural stabilization are tightly coupled to enzymatic activity [57, 58].

Based on these findings, we proposed a model in which EXD2 dynamically balances degradation and protection of RDHs through force- and damage-dependent switching (Fig. 7). Under high mechanical tension, such as during transcription or replication, EXD2 engages RDHs as a slow 3′-5′ exonuclease, trimming RNA in a concentration-dependent manner. In contrast, under low tension or in the presence of RNA damage, elevated local concentrations of EXD2 favor multimerization and co-condensation, resulting in compact and protective nucleoprotein assemblies. To validate this mechanistic model in cells, our *in vivo* revealed that EXD2 predominantly localizes to mitochondria. Crucially, our cellular experiments demonstrated that in areas of dense cell growth, individual cells experience intense crowding and severe mechanical compression, resulting in marked morphological deformation (Supplementary Fig. S16). Strikingly, the endogenous expression level of EXD2 in these compressed, deformed cells was significantly higher compared to cells maintaining a normal, sparse morphology. This phenomenon is highly consistent with established mechanobiological principles, wherein cell crowding-induced physical confinement and deformation elicit strong transcriptional reprogramming and replication stress, thereby triggering the upregulation of protective caretakers to safeguard genomic integrity. Under such crowding scenarios, the localized accumulation of stress-induced EXD2 effectively drives the protein concentration beyond the threshold required to activate its cooperative multimerization mode [59–62]. Upon overexpression, EXD2 redistributes to mitochondrial cristae and unexpectedly slows the resolution of mitochondrial RDHs in a dose-dependent manner. This cellular behavior directly supports our single-molecule observations. Through this concentration-dependent bifurcation between catalysis and condensation, EXD2 functions as a promiscuous moonlighting factor that integrates mechanical cues and substrate integrity to regulate the fate of RDHs for genome maintenance.

## Supplementary Material

gkag762_Supplemental_File

## Data Availability

The data supporting this article are available on Zenodo (https://doi.org/10.5281/zenodo.21451228).
