# Supplementary material for: Dual and opposing roles of the EXD2 exonuclease in the resolution of RNA–DNA hybrids
Source: Nucleic Acids Res. 2026 Jul 31;54(14):gkag762. doi: 10.1093/nar/gkag762 (PMC13425236; doi:10.1093/nar/gkag762)
Supplement: gkag762_Supplemental_File [file gkag762_supplemental_file.pdf]

Supplementary Materials

Dual and opposing roles of the EXD2 exonuclease in the resolution of RNA–DNA hybrids

Meng Hu[1,†], Yinghong Chen[2,3,†], Yanan Li[1,4], Xia Zhang[1], Yi Zhao[1], Xiaoxuan Song[1], Lishuang Chen[1], Zhiyun Ren[1,5,6], Yuting Zhang[1], Yali Mi[2], Cong Liu[7], Wei Li[2], Chao Liu[2,*], and Bo Sun[1,*]

[1]School of Life Science and Technology, ShanghaiTech University, Shanghai 201210, China
[2]Guangzhou Women and Children's Medical Center, Guangzhou Medical University, Guangzhou, Guangzhou 510623, China
[3]Department of Basic Medical Sciences, Shantou University Medical College, Shantou, Guangdong 515041, China
[4]College of Life Sciences, Xinyang Normal University, Xinyang, Jinan 464000, China
[5]CAS Center for Excellence in Molecular Cell Science, Shanghai Institute of Biochemistry and Cell Biology, Chinese Academy of Sciences, Shanghai 200031, China
[6]University of Chinese Academy of Sciences, Beijing 100049, China
[7]Interdisciplinary Research Center on Biology and Chemistry, State Key Laboratory of Chemical Biology, Shanghai Academy of Natural Sciences (SANS), Shanghai Institute of Organic Chemistry, Chinese Academy of Sciences, Shanghai 201210, China

*To whom correspondence should be addressed. Email: sunbo@shanghaitech.edu.cn
Correspondence may also be addressed to Chao Liu. Email: liuchsdu@163.com

[†]The first two authors should be regarded as Joint First Authors.

This file includes

Supplementary Figures S1 – S16
Supplementary Table S1
Supplementary References 1 – 3

Supplementary Figures

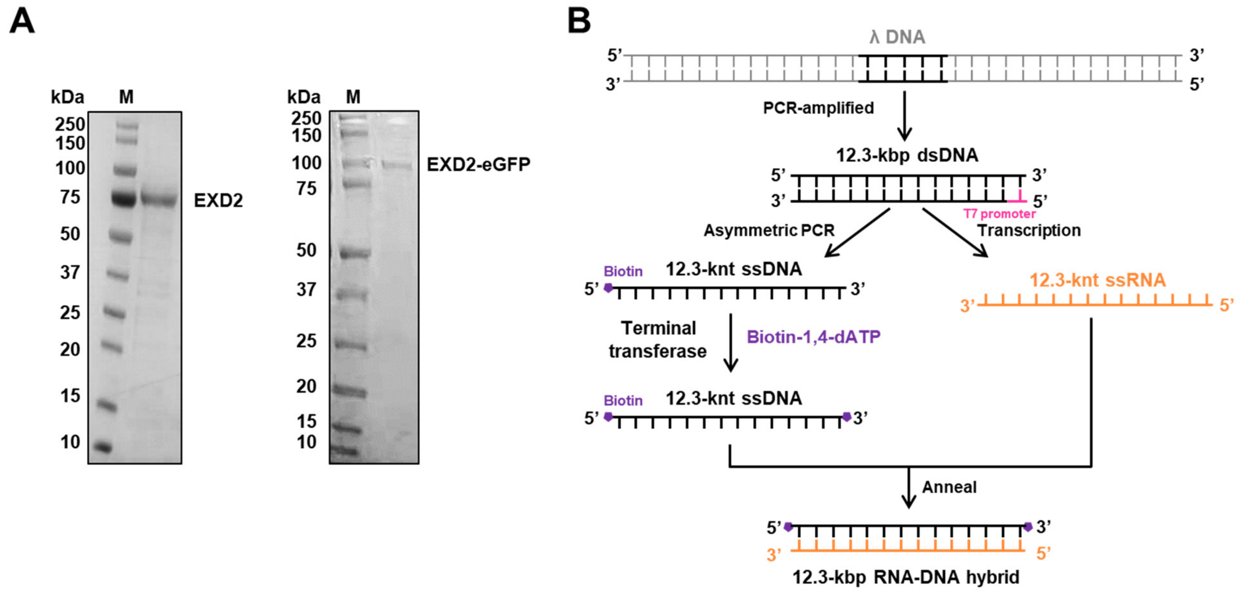

Figure S1. Protein purification and RDH template construction. (**A**) SDS-PAGE analysis of the purified proteins. Coomassie blue-stained gels demonstrate the purity of EXD2 (61-621) and EXD2-eGFP [1]. (**B**) dsDNA fragments of the desired length were amplified from λ DNA. Asymmetric PCR was performed using primers labeled with biotin or digoxigenin, followed by gel extraction and purification using a commercial kit (Thermo) to obtain single-stranded DNA. The 3' end of the ssDNA was further biotinylated using biotin-dATP (APExBIO) and terminal deoxynucleotidyl transferase. dsDNA fragments containing a T7 promoter were amplified from λ DNA to generate the corresponding single-stranded RNA by in vitro transcription. The 12.3-kbp hybrid template was assembled by annealing the ssDNA with its complementary RNA strand.

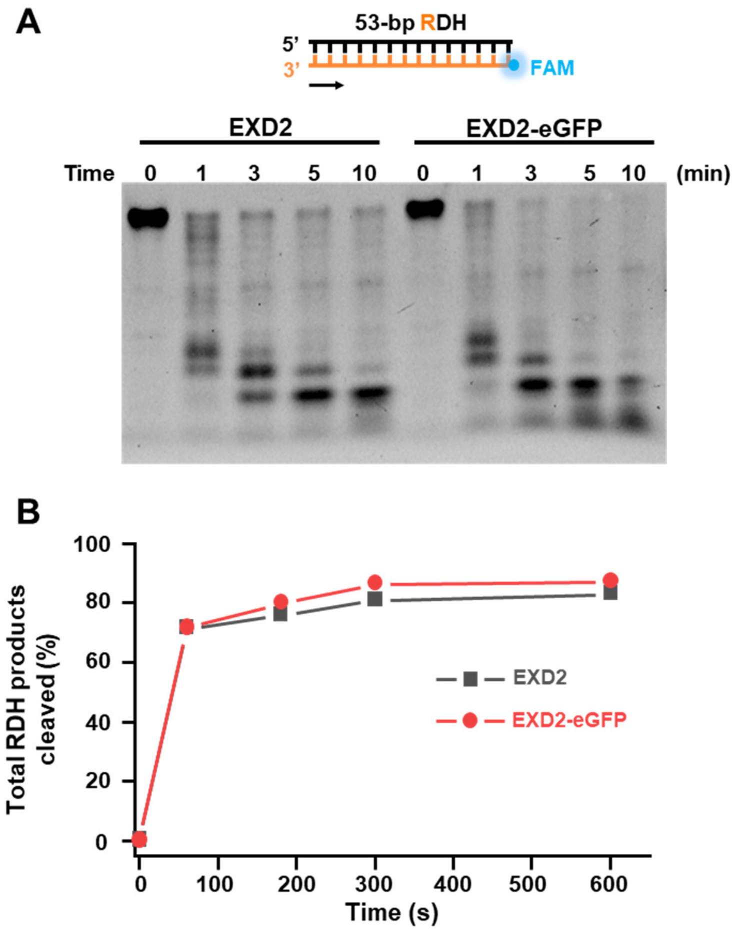

Figure S2. Validation of the catalytic activity of EXD2-eGFP. (**A**) A representative gel showing EXD2 and EXD2-eGFP (50 nM) cleaving the fluorescently labeled RDH (10 nM) substrates. The 5' end of the RNA strand is labeled with a FAM fluorophore (blue). The black arrow indicates the degradation direction. The cleavage products were resolved by an 12% denaturing polyacrylamide gel electrophoresis and visualized by phosphorimaging. The sequences of the substrates used are provided in Supplementary Table S1. (**B**) Quantification of the corresponding cleavage products as a function of time. Compared with the cleavage reactions triggered by EXD2 (black), the cleavage activities show almost no difference when initiated by EXD2-eGFP (red).

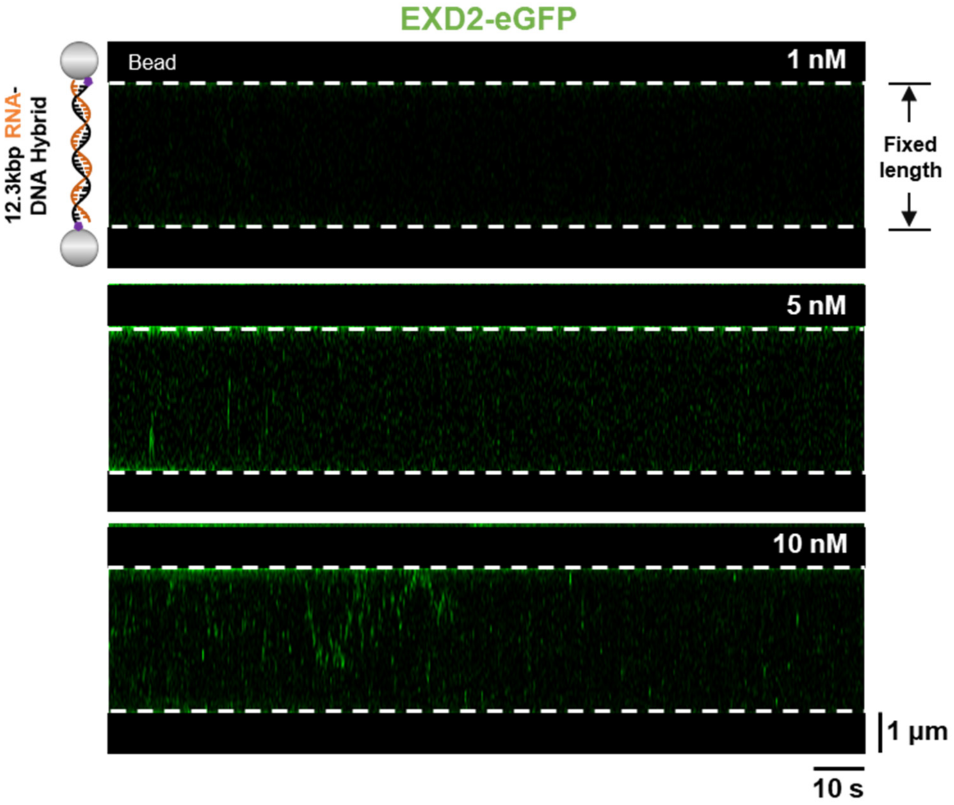

Figure S3. Detection of EXD2-eGFP binding to tensioned RDHs at low protein concentrations. kymographs showing EXD2-eGFP binding to a single 12.3-kbp RDH held at a fixed end-to-end distance of 3.6 µm in dual-trap optical tweezers. After stretching and fixing the RDH length, the tether was transferred into channels containing 1 nM, 5 nM, or 10 nM EXD2-eGFP, as indicated. Under all three conditions, no stable or discrete EXD2-eGFP binding events were detected along the RDH, indicating that EXD2 exhibits negligible association with extended RDHs at low protein concentrations.

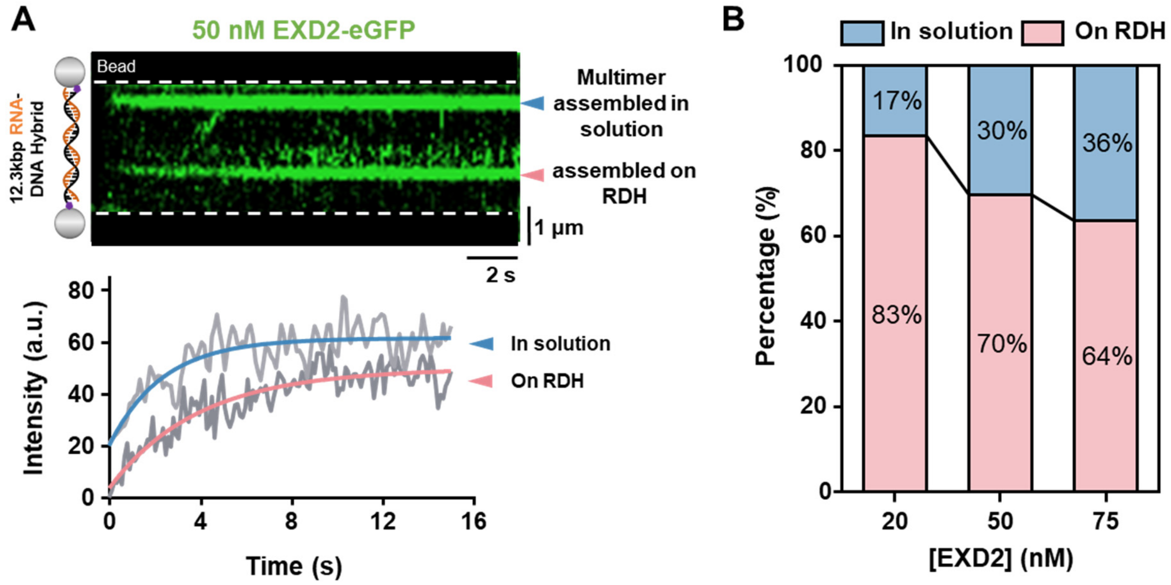

Figure S4. Pre-assembled EXD2 multimers directly bind onto RDHs. (**A**) kymographs of EXD2-eGFP binding to a single 12.3-kbp RDH held at a fixed end-to-end distance of 3.6 μm. After extension, the RDH was transferred into a channel containing 50 nM EXD2-eGFP. Two distinct binding behaviors were observed: EXD2 assemblies that formed progressively on the RDH after initial binding (pink arrowheads), and pre-assembled EXD2 multimers in solution that directly docked onto the RDH upon arrival (blue arrowheads). (**B**) Quantification of the relative contributions of progressive on-RDH multimer formation (pink) and direct docking of pre-assembled EXD2 multimers (blue) at 20 nM, 50 nM, and 75 nM EXD2-eGFP. The fraction of direct multimer docking events increases with protein concentration, indicating that EXD2 undergoes concentration-dependent self-multimerization in solution prior to RDH binding.

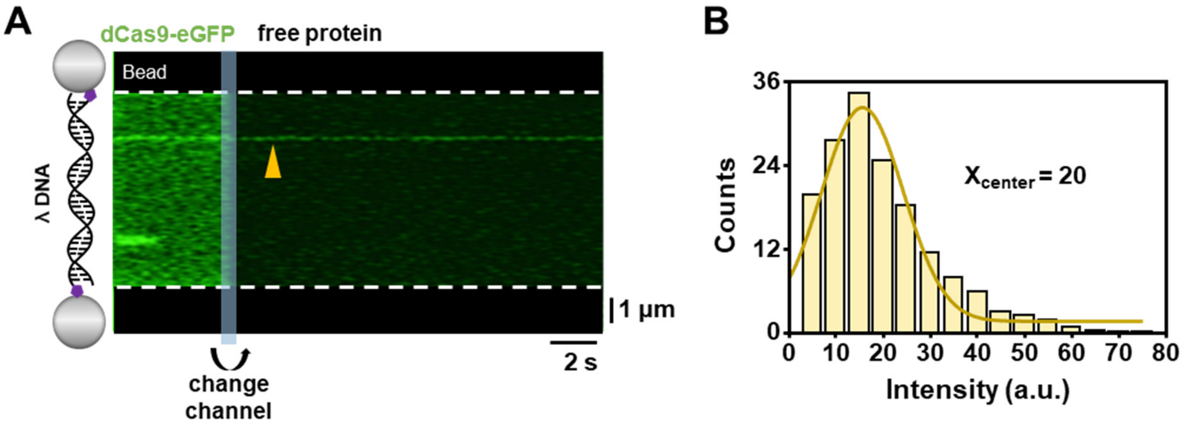

Figure S5. Calibration of single eGFP fluorescence intensity. (**A**) Representative fluorescence image of a single dCas9–eGFP/sgRNA complex bound to λ DNA under 5% laser power. The λ DNA tether was extended to a fixed length of 15 μm and transferred into a channel containing 10 nM dCas9–eGFP/sgRNA to allow binding. After individual binding events were observed, the tether was moved into protein-free buffer for fluorescence measurement [2]. (**B**) Gaussian fit of the fluorescence intensity distribution of single eGFP molecules (n=36). The fitted median fluorescence intensity (X_{center}) is 20, which was used as the reference value for single-eGFP calibration in subsequent stoichiometric analyses.

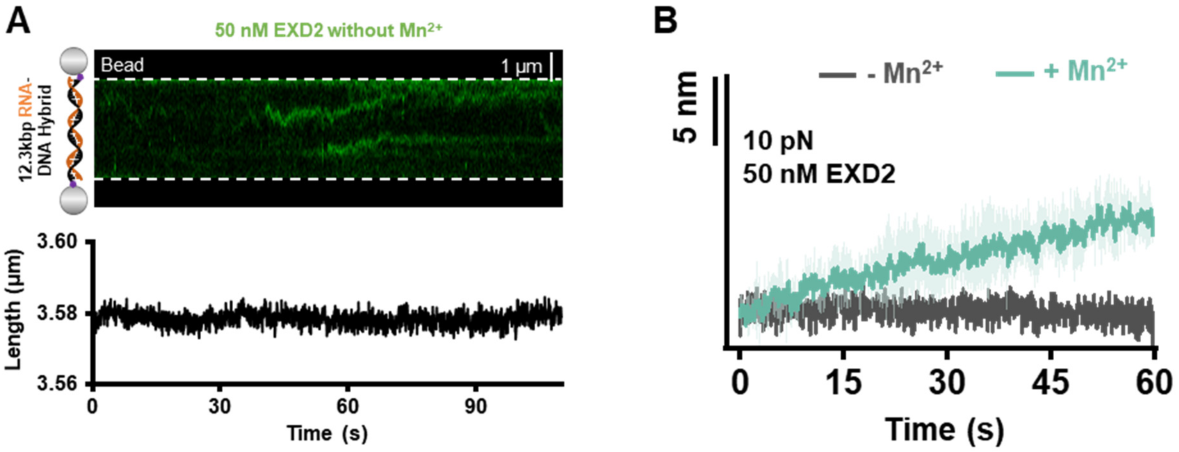

Figure S6. Hybrid extension stems from EXD2-mediated RDH digestion. (**A**) Representative kymograph and length trajectory of a single RDH molecule at 10 pN in a channel containing 50 nM EXD2-eGFP without Mn^{2+}. The stable tether length indicates that EXD2 remains bound to the substrate but cannot catalyze degradation in the absence of Mn^{2+}. (**B**) Representative time traces showing tether extension as a function of time at 10 pN under 50 nM EXD2 in the absence or presence of Mn^{2+}. In the presence of Mn^{2+}, a clear increase in tether length was observed (n=3), whereas no detectable extension change occurred when Mn^{2+} was omitted.

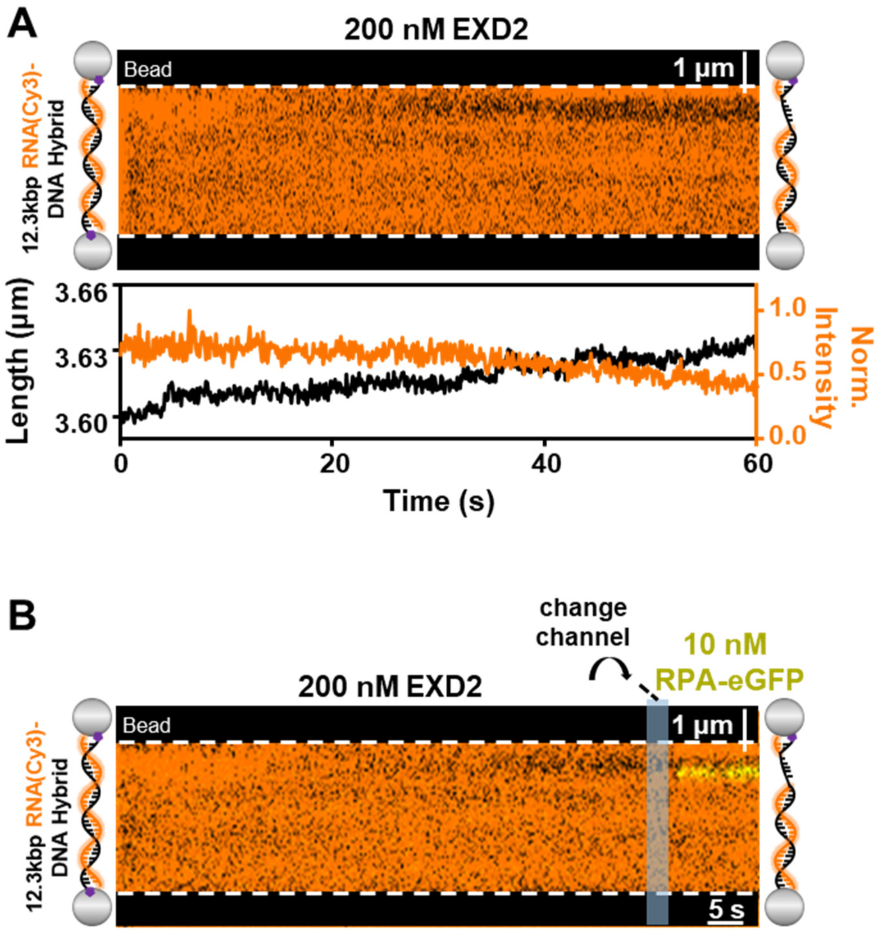

Figure S7. Real-time visualization of RNA degradation within RDHs by EXD2. (**A**) A 12.3-kbp RDH containing Cy3-UTP–labeled RNA was stretched at 10 pN and transferred into a channel containing 200 nM EXD2. The upper panel shows a representative kymograph in which progressive loss of Cy3 fluorescence generates dark gaps along the hybrid, indicating RNA degradation. The lower panel plots the RDH extension and total RNA fluorescence intensity as a function of time, revealing a concomitant increase in tether length and decrease in RNA signal. (**B**) Representative kymograph showing an RNA(Cy3)-DNA Hybrid incubated in a channel containing 200 nM EXD2 under a constant force of 10 pN for 80 s. The tether was subsequently transferred into a channel containing 10 nM RPA-eGFP. Single-stranded DNA generated by EXD2-mediated cleavage recruited RPA-eGFP, which appears as yellow–green fluorescence along the tether.

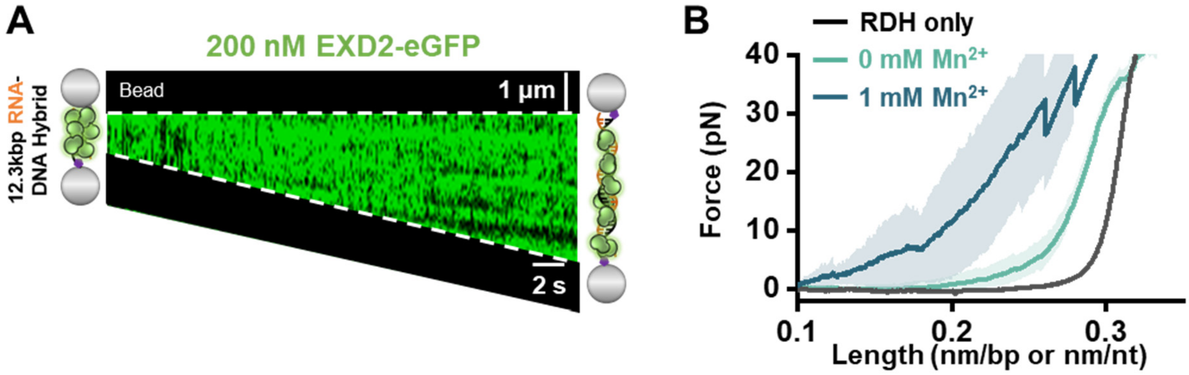

Figure S8. Depletion of Mn^{2+} attenuates EXD2-mediated RDH condensation. (**A**) Representative kymograph illustrating the stretching of an EXD2-bound RDH in the absence of Mn^{2+}. The RDH was first relaxed by actively moving the steerable bead toward the fixed trap to allow EXD2 binding under low tension. The tether was then stretched by separating the traps at a constant speed of 0.1 µm/s. (**B**) Representative force–extension curve of an RDH bound by EXD2 oligomers under Mn^{2+}-depleted and 1 mM Mn^{2+} conditions. The black trace denotes the canonical force–extension behavior of a naked RDH in the absence of EXD2. The reduced contour length of the EXD2-bound RDH indicates that shortening arises from EXD2-mediated condensation.

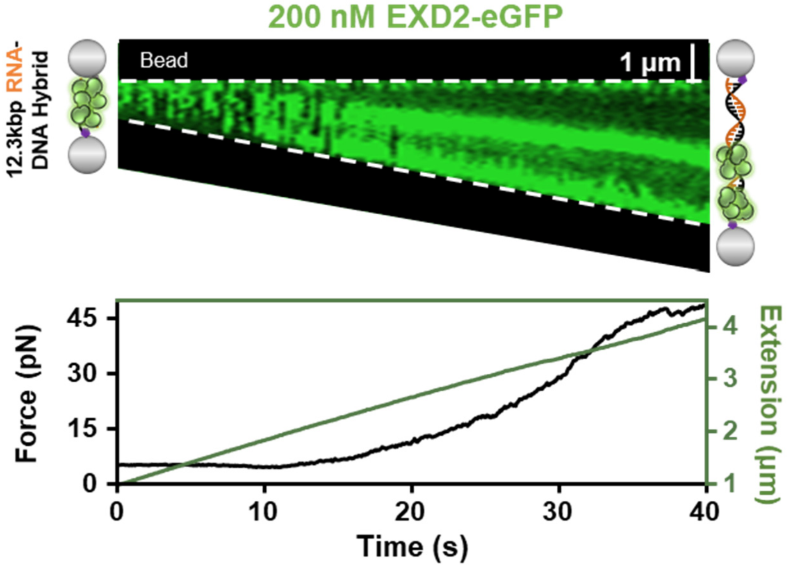

Figure S9. EXD2 micro-condensates are maintained during RDH stretching. A representative kymograph of RDH showing the stretching of RDH bound by EXD2-eGFP multimers. The corresponding force and extension of the DNA are shown below the kymograph. Throughout stretching, EXD2 fluorescence puncta remained stable.

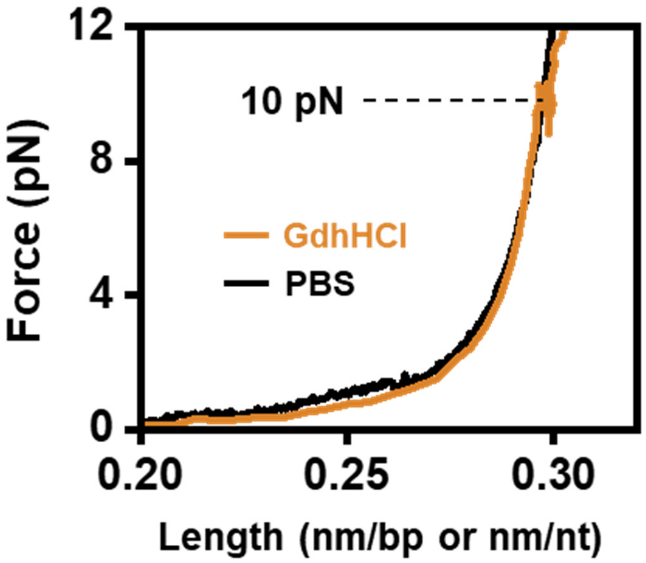

Figure S10. GdhHCl has minimal impact on RDH elasticity below 10 pN. Comparison of force-length curves in PBS (black) and GdhHCl-containing buffer (orange). The overlap of the profiles at forces below 10 pN confirms that the denaturant does not alter the intrinsic mechanical properties of the substrate under the experimental conditions used for protein binding.

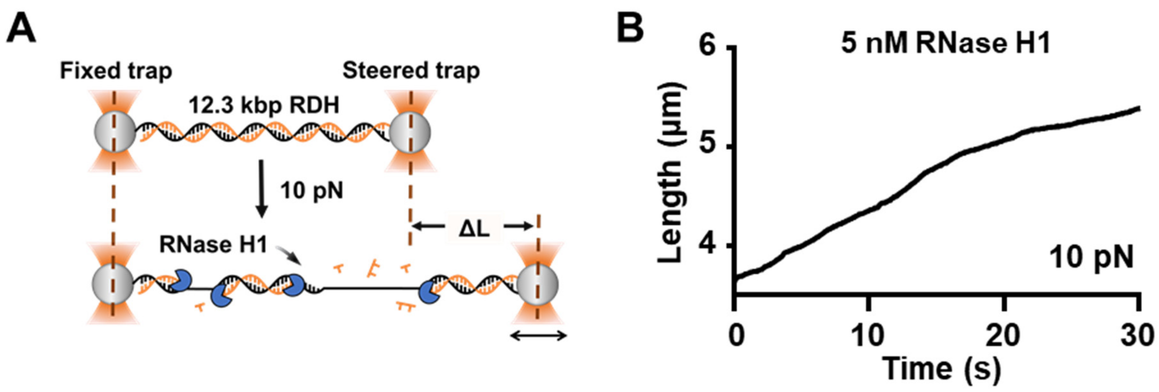

Figure S11. RNase H1 digests tensioned RDHs at a fast rate. (**A**) Model shows a schematic of the dual-trap optical tweezers assay used to monitor RNase H1-mediated processing of single RDH molecules. (**B**) Naked 12.3-kbp RDH was incubated in a 5 nM RNase H1 channel at 10 pN for 30 seconds.

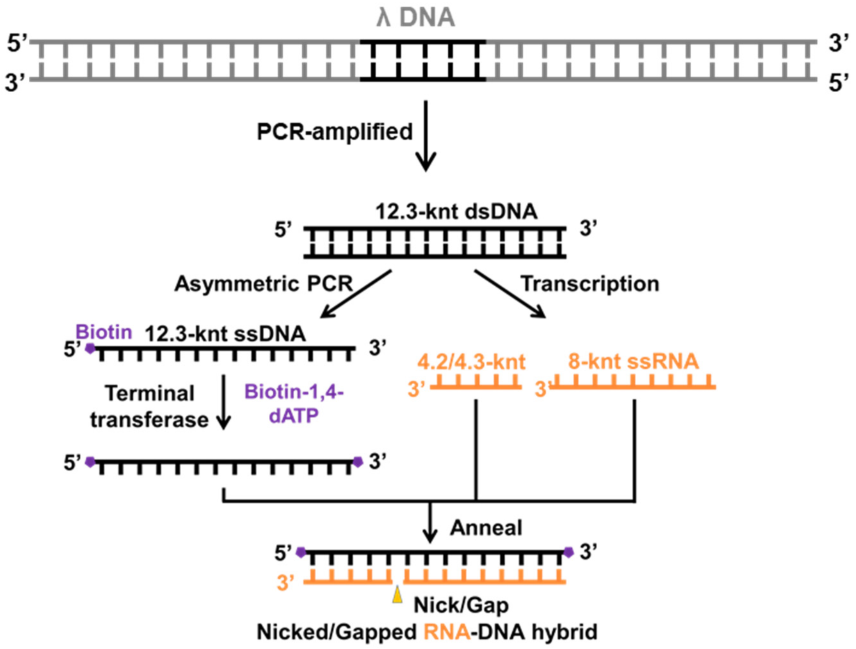

Figure S12. Design of the nicked and gapped DNA templates. To generate nicked/gapped RDHs, two ssRNA transcripts (8 and 4.2/4.3 kb) were annealed to a 12.3-kb ssDNA backbone, following standard hybrid assembly procedures.

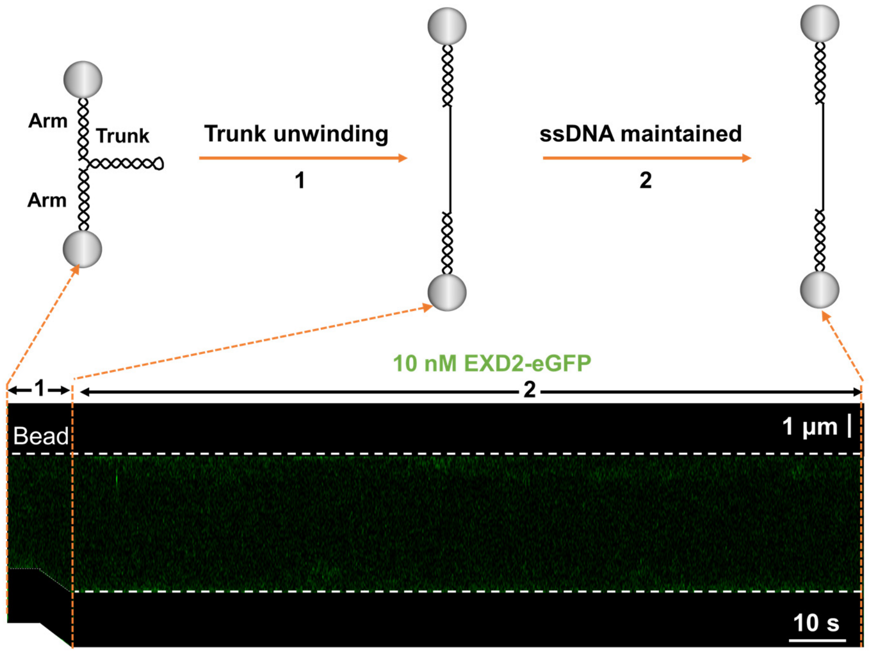

Figure S13. EXD2 exhibits minimal binding to ssDNA. A representative kymograph of the T-shaped DNA template and its corresponding DNA length in the presence of 10 nM EXD2-eGFP under 15 pN [3]. The top schematic illustrates the experimental workflow for generating a single-stranded DNA (ssDNA) template from a dsDNA trunk structure. Initially, the optical trap was manually steered to unwind the dsDNA trunk until a full-length ssDNA tether was produced (Step 1). Subsequently, a constant force of 15 pN was maintained to ensure the ssDNA remained extended without re-forming the trunk structure. The resulting ssDNA template was then transferred into a microfluidic channel containing 10 nM EXD2-eGFP (Step 2).

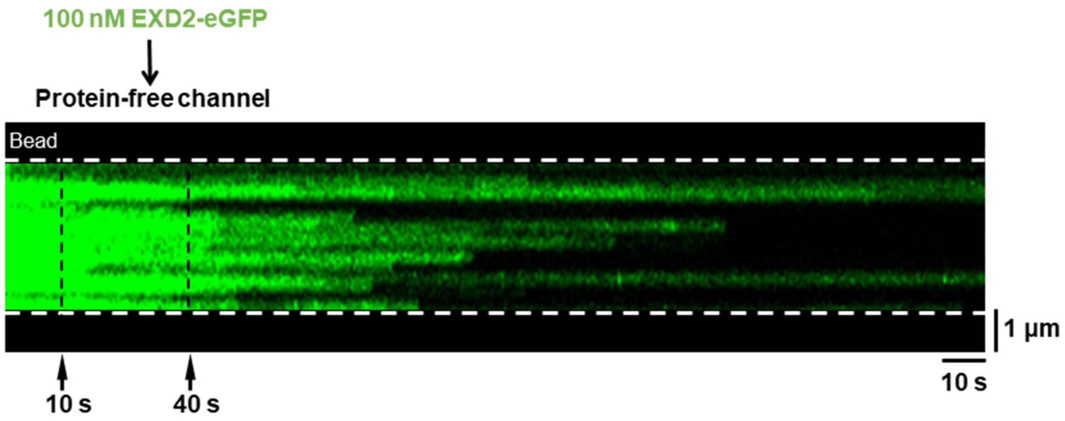

Figure S14. Quantification of fluorescence signals of RDH-bound EXD2-eGFP. Representative kymographs of EXD2-eGFP binding on RDH at 100 nM concentrations. After 30 seconds of incubation in the protein channel, RDH was moved to the buffer channel and imaged with 5% laser power (0.5 W).

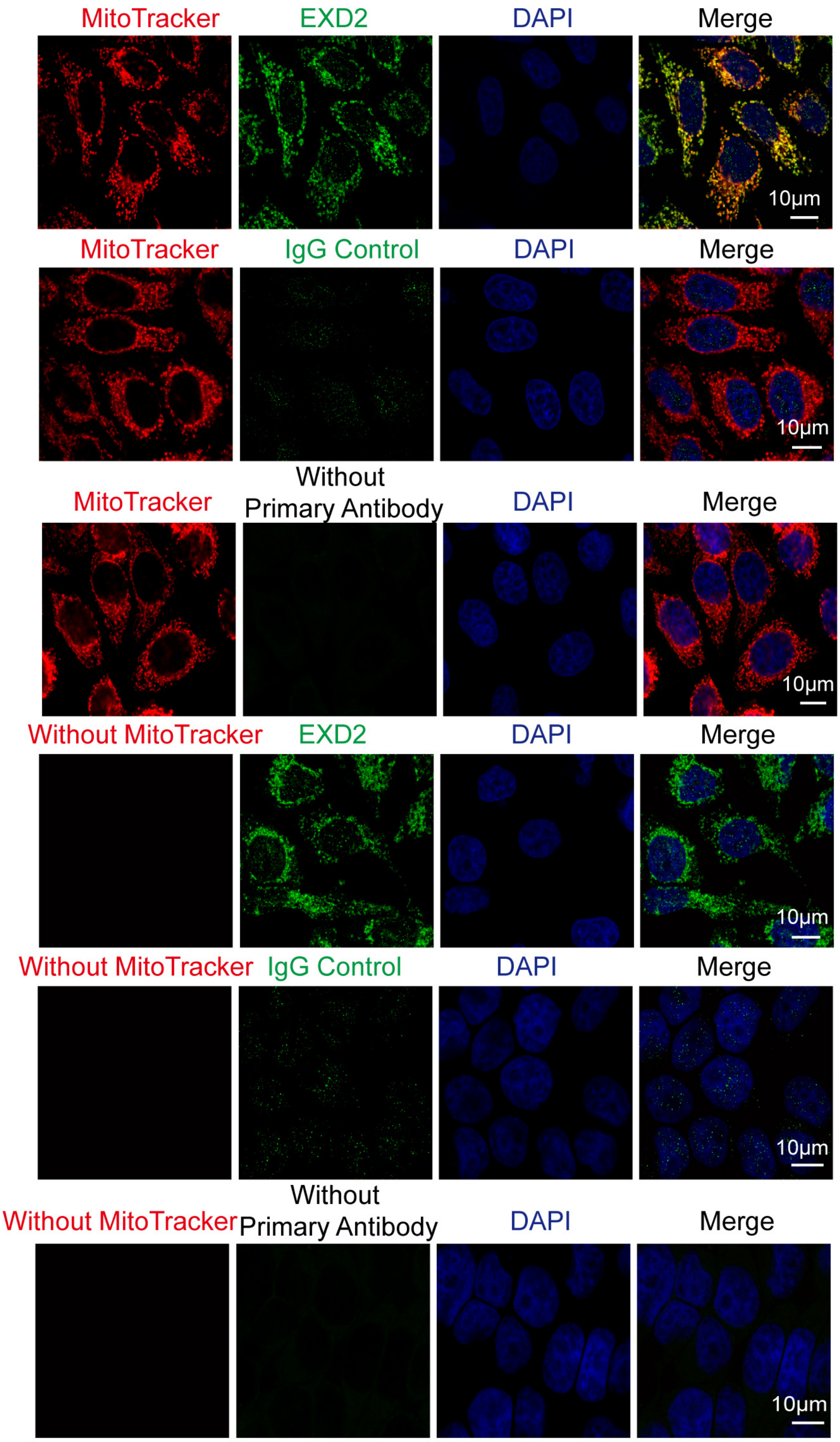

Figure S15. Endogenous EXD2 was predominantly localized to the mitochondria. Representative immunofluorescence images of EXD2 (green) and MitoTracker (red) in HeLa cells. Nuclei were stained with DAPI (blue). These observations support that endogenous EXD2 was predominantly localized to the mitochondria.

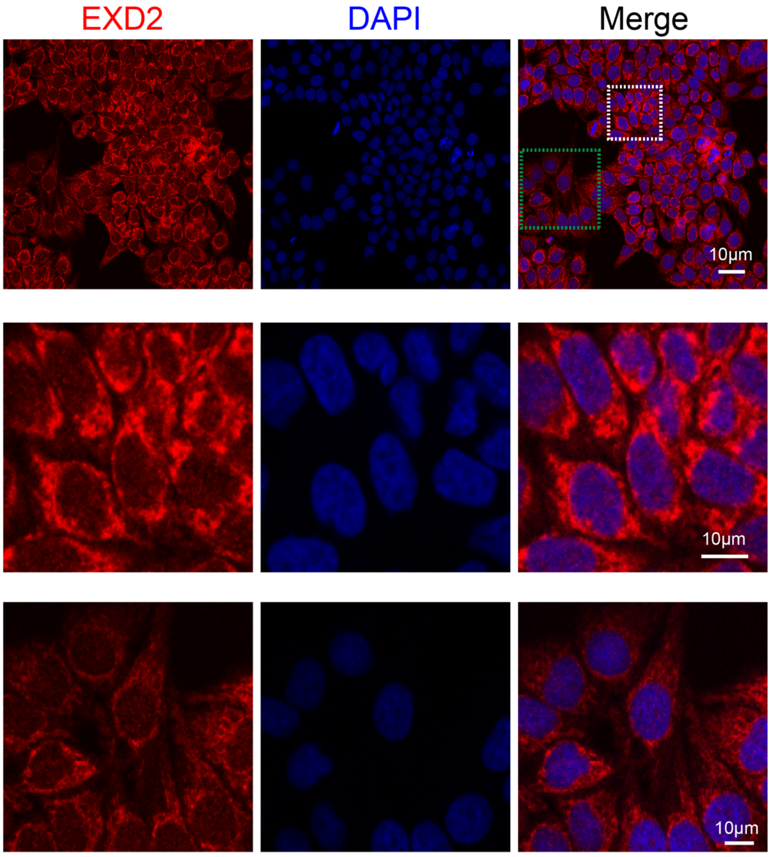

Figure S16. Cell crowding and mechanical compression upregulate endogenous EXD2 expression. Global immunofluorescence images (top row) show endogenous EXD2 (red) and nuclei (DAPI, blue) in HeLa cells cultured at heterogeneous densities, with a highly crowded region under intense mechanical constraint outlined by a white dashed box and a sparsely populated control area outlined by a green dashed box. Magnified views of the crowded region (middle row) reveal that cells subjected to physical confinement exhibit pronounced morphological compression and deformation, accompanied by a robust and significant elevation in endogenous EXD2 fluorescence intensity. Conversely, magnified views of the uncrowded control region (bottom row) show relaxed, normal cellular morphology with baseline levels of endogenous EXD2 expression. Scale bars: 10 μm for all panels.

Supplementary Table 1. Sequences of primers and oligonucleotides used.

Template	Name	Sequence
12.3-kbp dsDNA	forward	5′-AGCCTTTGCCTCGCTATACA-3′
	reverse	5′-CAGCATAAGCGGCTACATGA-3′
12.3-kbp dsDNA for transcription	forward	5′-TAATACGACTCACTATAGGAGCCTTTGCCTCGCTATACA-3′
	reverse	5′-CAGCATAAGCGGCTACATGA-3′
12.3-kbp ssDNA	forward	5′-biotin-CAGCATAAGCGGCTACATGA-3′
8-kbp dsDNA for transcription	reverse	5′-CAGCATAAGCGGCTACATGA-3′
	forward	5′-TAATACGACTCACTATAGGAGCCTTTGCCTCGCTATACA-3′
4.3-kbp dsDNA for transcription	reverse	5′-AGGTGCAGTACAGCATGTGGGTGACGCGA-3′
	forward	5′-TAATACGACTCACTATAGGGCCATGTAAGCTGACTTTATGGC-3′
53-bp RDH-FAM	DNA	5’-TGTAGTTGCCGTCGTCCTTGAAGAAGATGGTGATCTCCTGGACGTAG CCTTCG-3’
	RNA	5’-FAM-CGAAGGCUACGUCCAGGAGAUCACCAUCUUCUUCAAGGACG ACGGCAACUACA-3’

Supplementary References

1. Jia, X., Li, Y., Wang, T., Bi, L., Guo, L., Chen, Z., Zhang, X., Ye, S., Chen, J., Yang, B. *et al.* Discrete RNA-DNA hybrid cleavage by the EXD2 exonuclease pinpoints two rate-limiting steps. *EMBO J* 2023; **42**: e111703. https://doi.org/10.15252/embj.2022111703.

2. Zhao, Y., Guo, L., Hu, J., Ren, Z., Li, Y., Hu, M., Zhang, X., Bi, L., Li, D., Ma, H. *et al.* Phase-separated ParB enforces diverse DNA compaction modes and stabilizes the parS-centered partition complex. *Nucleic Acids Res* 2024; **52**: 8385–8398. https://doi.org/10.1093/nar/gkae533.

3. Wang, T., Hu, J., Li, Y., Bi, L., Guo, L., Jia, X., Zhang, X., Li, D., Hou, X.M., Modesti, M. *et al.* Bloom Syndrome Helicase Compresses Single-Stranded DNA into Phase-Separated Condensates. *Angew Chem Int Ed Engl* 2022; **61**: e202209463. https://doi.org/10.1002/anie.202209463.
